# Ancient microbial DNA and proteins preserve in concretions covering human remains

**DOI:** 10.1016/j.isci.2025.113182

**Published:** 2025-07-23

**Authors:** Biancamaria Bonucci, Toni de-Dios, Rémi Barbieri, Jess Emma Thompson, Sofia Panella, Francesca Radina, Sandra Sivilli, Helja Kabral, Anu Solnik, Mary Anne Tafuri, John Robb, Christiana Lyn Scheib

**Affiliations:** 1 Estonian Biocentre, Institute of Genomics, University of Tartu, Riia 23B, 51010 Tartu, Estonia; 2 McDonald Institute for Archaeological Research, University of Cambridge, Downing Street, Cambridge CB2 3ER, UK; 3 Department of Environmental Biology and Mediterranean bioArchaeological Research Advances (MAReA) Centre, Sapienza University of Rome, Piazzale Aldo Moro 5, 00185 Rome, Italy; 4 Soprintendenza Archeologia, Belle Arti e Paesaggio per la Città Metropolitana di Bari, Corso Antonio De Tullio, 2, 70122 Bari, Italy; 5 Independent Researcher, Bari, Italy; 6 Core Lab Facility, Institute of Genomics, University of Tartu, Riia 23B, 51010 Tartu, Estonia; 7 Department of Zoology, University of Cambridge, Cambridge CB2 3EJ, UK; 8 St John’s College, University of Cambridge, Cambridge CB2 1TP, UK

**Keywords:** Microbiome, Omics, Proteomics, Archeology

## Abstract

Archaeological remains covered with concretions, including human bones, are commonly found in certain areas and time periods of interest for understanding the past, but have yet to be investigated for potential ancient DNA (aDNA) and protein content. We extracted aDNA and proteins in tandem from human dental remains and their surrounding concretions and compared them to non-concreted human dental remains from the same site. Concretions appeared homogeneous in color and texture, consisting of a hard dark gray sediment adhered to the bone surfaces, presumably as a result of cyclical waterlogging of the burial deposits. Concretions were found to contain human oral microbial genomes and proteins, probably leached from the original skeletal source, as well as environmental and human proteins. Despite this, both the original teeth and the concretions surrounding them lacked endogenous human aDNA, indicating that the use of this type of material in future molecular archaeological applications is limited.

## Highlights

• Sediment concretions found tightly adhered to human remains from Neolithic Puglia
• Concreted tissues display poor human aDNA preservation
• Concreted tissues can contain high-quality ancient microbial genomes
• Ancient DNA and proteins from the same oral microbiome species can be recovered

## Graphical abstract

**Figure:**
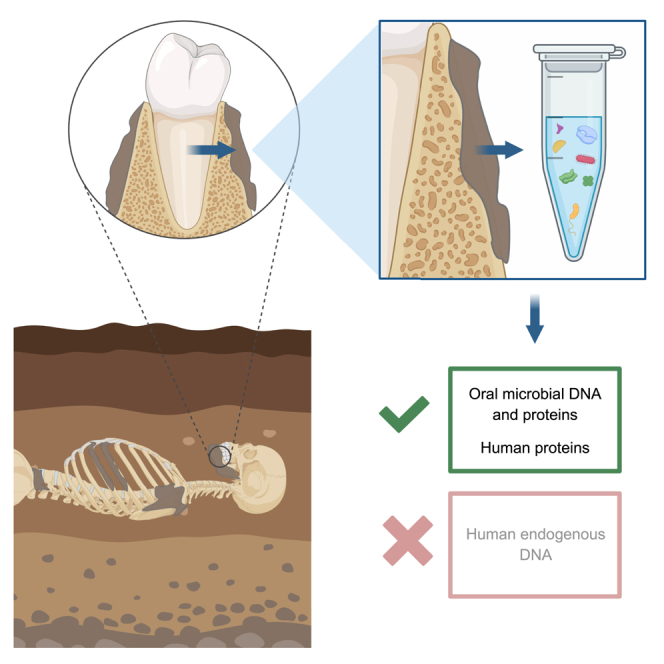

## Introduction

Human dental and skeletal tissues are the primary sources of ancient DNA (aDNA) molecules, providing valuable information about past population dynamics,^1^^,^^2^ lifestyle,^3^^,^^4^^,^^5^ and infectious diseases.^6^^,^^7^ These discoveries have not only enriched our understanding of human evolutionary history but have also shed light on the genetic diversity and interbreeding events among different hominin groups.^8^^,^^9^^,^^10^^,^^11^ Recent advances in aDNA methods have expanded the range of alternative substrates from which aDNA and proteins can be recovered. The presence of ancient human biomolecules on certain substrates can be attributed to various factors, including direct contact with human hard or soft tissues or the transfer of human aDNA through different processes. These processes may involve chewing, wearing specific materials, or habitation activities. Such materials previously investigated include archaeological dental calculus,^12^^,^^13^^,^^14^ mummies,^15^ calcified tissues,^16^^,^^17^ paleofaeces,^18^^,^^19^^,^^20^ materials which came into contact with human bodies/skin,^21^ cave stones,^22^ and sediments.^22^^,^^23^^,^^24^

In addition to human DNA, these substrates have also yielded valuable information about ancient microbial communities. Metagenomic analyses of these samples have revealed the presence of various pathogens, commensal microbes, and environmental microorganisms, offering insights into ancient disease transmission, dietary habits, and environmental conditions.^20^^,^^25^^,^^26^^,^^27^ Moreover, the analysis of proteins extracted from sediment and dental calculus have provided complementary evidence about ancient diets, food processing techniques, and the presence of specific biomarkers indicative of health and disease.^12^

Key advancements in the field have therefore provided the opportunity to study previously overlooked materials. For example, it has been shown that calcite concretions formed from stalactites can be considered not only mineralogical deposits, but also an extension to the archaeological remains as in some cases they may retain and preserve information in the form of aDNA.^22^

**Figure 1:**
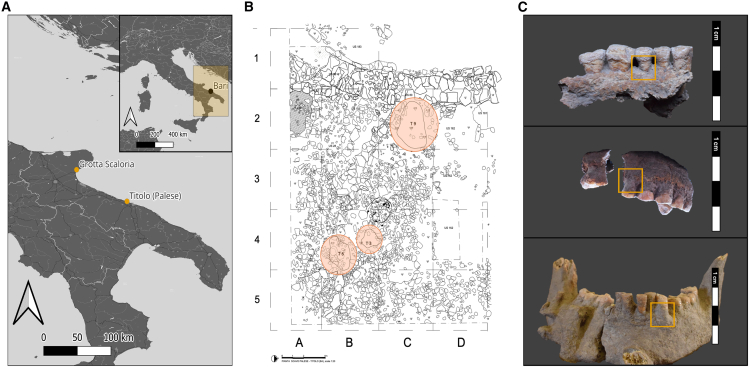
Map of Italy, with the sites highlighted, site map, and archaeological material (A) Map of Italy and detail of Puglia, displaying the sites named in text. (B) Layout of burials at Titolo (Palese, BA, Puglia, Italy) with the location of the three burials with skeletal remains covered by sediment concretions highlighted in orange. (C) Concretion-covered dentitions of the three individuals under study: PAL002, burial T3 (top), PAL005, burial T8 (middle), PAL006, burial T9 (bottom), with sampled region in yellow.

Here, we report analyses carried out on concreted and non-concreted human dental and skeletal remains from Titolo, a large Neolithic village located within the modern neighborhood of Palese-Macchie (Bari, Puglia, Italy) (Figure 1A). Titolo was occupied from the early 6th millennium BCE and into the late Neolithic, with burials predominantly dating to the middle Neolithic (mid-6th millennium BCE). The settlement is located on the highest part of a promontory, and is characterized by monumental stone wall structures, the remains of huts and other small structures in earth, clay, and stone, and a series of burial pits containing primary and secondary depositions (Figure 1B). The burials were deposited over a layer of *terra rossa* soils just above the limestone bedrock (Sivilli S. 2018, unpublished). Terra rossa is a reddish, clayey or silty clayey soil, characterized by high internal drainage and neutral pH condition.^28^ The majority of the skeletal remains were almost completely covered by sediment concretions, consisting of a hard gray sediment adhered to the bone surfaces, which were presumably formed by fluctuating precipitation and aridity affecting the limestone-rich soils (Figure 1C). Archaeologists had tried to remove concretions post-excavation by using a combination of diluted hydrochloric acid and scraping; this was evident from large areas of scrape marks and striations across bone surfaces. In order to explore concretion-covered tissues as a source to extract ancient microbial communities, human aDNA and protein content from, we subsampled the dentitions of individuals T3, T8, and T9 (Figure 1C, Table 1). For two individuals (T8 and T9), we sampled the whole tooth root, a chunk of the concretion attached to the alveolar bone around the root, and sediment powder from nearby on the dentition, while for T3 we could not sample the concretion chunk (only powder) (Figure S1). All sub-samples and two calculus samples from the same site were processed for aDNA and protein recovery and sequenced using next generation sequencing (NGS) technology and liquid-chromatography mass spectrometry (LC-MS/MS) (Table 1). The results obtained by the aDNA extraction were compared to 7 non-concreted tooth roots and 9 dental calculus from 4 individuals from Titolo. As cave environments typically provide excellent conditions for DNA preservation,^29^ we carried out analysis on 38 post-cranial remains from the contemporary disarticulated deposit of Grotta Scaloria, a large cave located in Manfredonia (Foggia, Puglia, Italy) (see Supplementary material for detailed archaeological context). The preservation in the cave appeared to be very low, so we excluded the site from subsequent analysis, but we included the results in the supplementary tables.

**Table 1:** Summary information for samples with both aDNA and protein data

Sample ID	Burial ID	Weight (mg)	Sample	Element
PAL002F	T3	247.0	Tooth Root	URP2
PAL002G	T3	2.7	Concretion Powder covering element	
PAL005D	T8	110.0	Tooth Root	URI2
PAL005E	T8	6.0	Concretion Powder covering element	
PAL005F	T8	17.9	Chunk of Concretion attached to the element	
PAL006B	T9	173.0	Tooth Root	LRI2
PAL006C	T9	26.6	Chunk of Concretion attached to the element	
PAL006D	T9	21.8	Concretion Powder covering element	
PAL001A	T2	2.0	Calculus	LLI1
PAL005A	T8	2.0	Calculus	ULC

Sample ID is the ID assigned in the UTIG lab. Burial ID refers to the archaeological context. The last column indicates the tooth from which the sample was taken, namely URP2 (upper right premolar 2), URI2 (upper right incisive 2), LRI2 (lower right incisive 2), LLI1 (lower left incisive 1), ULC (upper left canine).

## Results

### Initial sample processing and ethics

Ancient DNA and protein extraction are destructive procedures for the remains being studied. This research adhered to internal project and laboratory guidelines, which included thoroughly documenting and photographing the remains before any samples were taken. To ensure minimal destruction, most samples included in this study were obtained from larger samples and used for combined analyses. All necessary permits for the described study were acquired, ensuring compliance with all relevant regulations.

We produced 24 libraries from 24 total samples from 5 individuals from the site of Titolo. In addition, we generated data for 38 disarticulated, commingled post-cranial remains from the contemporary site of Grotta Scaloria (See supplementary materials). All samples were sequenced to a screening depth of approximately 20 million reads on NextSeq550 (Illumina), accounting for a total of 503,449,829 number of reads generated (see STAR Methods). All libraries were mapped against the human reference genome and screened using KrakenUniq.^30^

### Low ancient human DNA content in concretions and corresponding tissues

**Figure 2:**
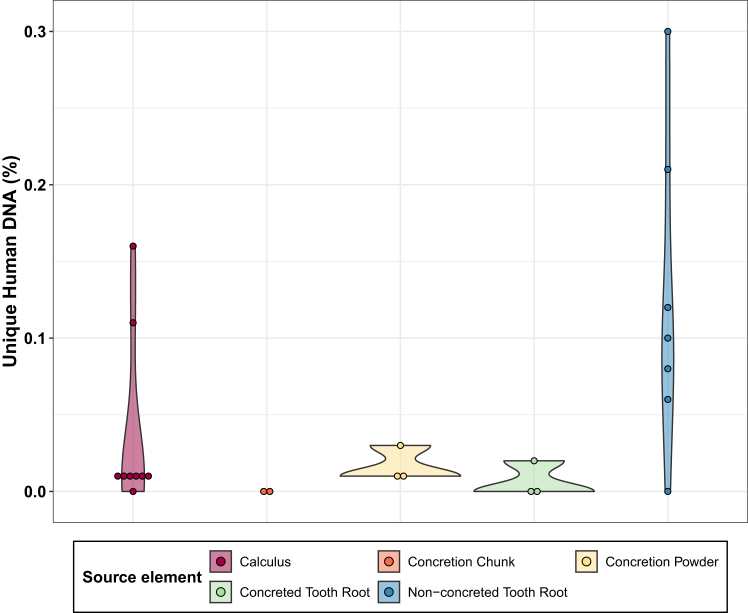
Percentage of unique human DNA per sample type Shown are the tested concretions, calculus, and non-concreted tooth roots from the site of Titolo.

Initial analysis across all Titolo samples revealed low preservation of human DNA with values ranging from 57 to 3646 reads (<0.03% of human DNA content) in the test set and 375–26502 reads (0.01%–0.30% human DNA content) in the control set (Figure 2; Table S2). However, the low deamination rates detected in some of the samples may indicate modern contamination, possibly originating from sample handling that could not be removed by the procedures adopted in the clean lab.

The remaining reads were identified by KrakenUniq as belonging to microbial (2%–6.8% test set; 0.92%–4.8% control set) or were unclassified (93%–98% test set; 95.16%–98.82% control set) (Tables S3 and S4). We filtered our data to include only those species assignation with an E-score above 7, which is the minimum threshold we used to trust that the assignation of a microbe is true (see STAR Methods). Both screenings of human and especially microbial genomes reveal poor aDNA preservation for Grotta Scaloria samples, therefore we could not proceed with further analysis, but we report the results of the mapping to the human genome and metagenomic screening with KrakenUniq, respectively, in Tables S2 and S4, and we describe the archaeological background in the Supplement text.

### Oral microbiomes from concretions are comparable with literature data

**Figure 3:**
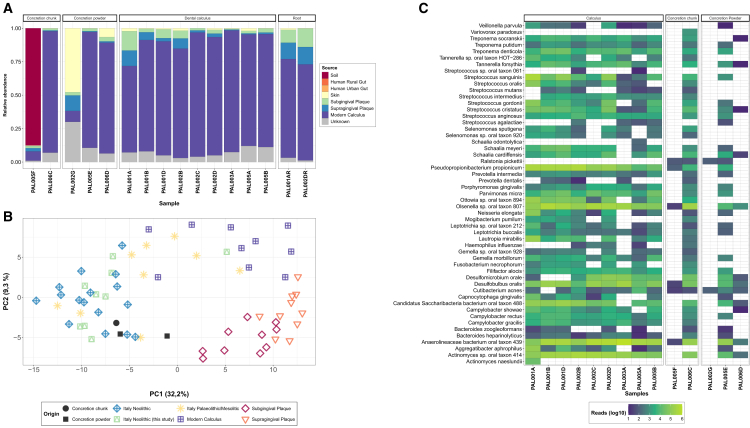
Microbial ecology background of the samples (A) Stacked bar plot of Sourcetracker analysis showing the proportion of KrakenUniq classified reads at the species level stemming from modern dental calculus, modern subgingival and supragingival plaque, skin, soil, and gut microbiome (both rural and urban) in the samples taken into consideration in our study. (B) PCA of samples in context of modern and ancient published oral metagenomes.^32^^,^^33^ (C) Heatmap of oral species detected. The number of reads is displayed in log10 scale. All E-scores are over 7.

Within the microbial data, we used SourceTracker2^31^ to identify the likely sources of species present in each sample (Figure 3A; Table S5, S6). The samples shown are those which contained enough reads to perform microbial source tracking (see STAR Methods). The analysis of the 9 dental calculus samples from Titolo showed that 55% to 95% of the classified reads originated from oral taxa, while very few stemmed from the skin, soil, or gut microbiome. From the test samples, PAL002G and PAL005F showed little presence of oral microbes, while much of the microbial species present in PAL005E, PAL006C, and PAL006D (from 50% to 80%) belonged to a human oral source (Figure 3A). To investigate this further, we used principal-component analysis (PCA) to place the ancient oral microbial ecology in relation to modern and ancient published samples (Figure 3B). All the dental calculus from this study and samples PAL005E, PAL006C, and PAL006D appeared to be comparable to other ancient and modern published^32^^,^^33^^,^^34^ dental calculus samples. The plot of PCA loadings (Figure S2) indicates that Anaerolineaceae bacterium oral taxon 439 (henceforth indicated as Abot439), Olsenella sp. oral taxon 807 (Osot807) and *Desulfomicrobium orale* are the species that most contributed to the distribution of the samples in the left quadrant of the PCA plot.

We further analyzed the content of oral bacteria in the dental calculus from our study and in the test samples by quantifying the number of reads assigned to oral species found in a custom dataset generated by merging the SourceTracker2 results and the human oral microbiome database (eHOMD)^35^ (see STAR Methods) (Figure 3C). For the dental calculus, we detected the unequivocal presence of common oral bacteria and potential oral pathogens such as *Tannerella forsythia*, *Treponema denticola*, *Porphyromonas gingivalis*, and *Desulfomicrobium orale*, among others. Despite the fact that the concretions seem to show generally lower abundances of oral bacteria, PAL005E and PAL006C yielded a substantial number of reads for 33 and 46 oral species, respectively. In particular, we found a high abundance of two sulfate-reducing bacteria, *Desulfobulbus oralis* and *Desulfomicrobium orale,* as well as other common oral species such as Abot439, Actinomyces sp. oral taxon 414 (Asot414) and Osot807.

### Whole ancient oral microbial genomes retrievable from concretions

**Figure 4:**
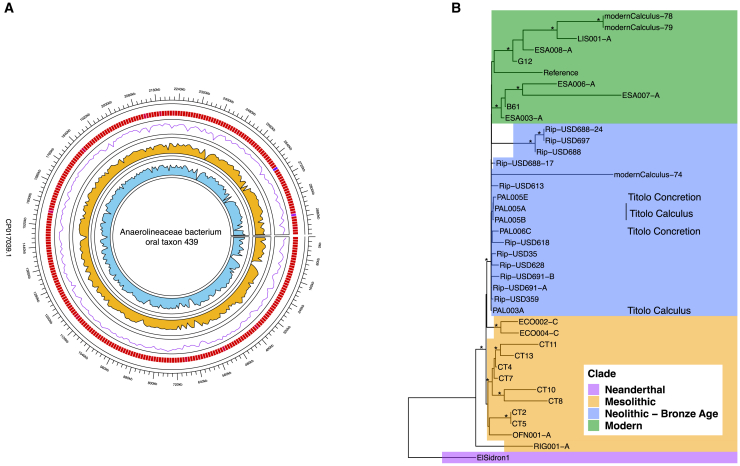
Circos plot of the mapping of samples PAL005E and PAL006C and phylogenetic tree of Abot439 (A) Circos coverage plot mapping samples to Abot439 reference genome. From the outer to the inner ring: mappability, GC content, depth of coverage for sample PAL005E, depth of coverage for PAL006C. (B) Maximum-likelihood tree created with RAxMl and 1,000 bootstraps. The tree includes 16,469 biallelic positions and 39 modern and ancient Abot439 strains. Clades are highlighted in color. Nodes with a support over 90% are marked with an asterisk (∗).

In order to investigate the presence of oral bacteria in concreted tissues, we explored in more detail the most prevalent species in PAL005E and PAL006C, by aligning the sequences from PAL005E against the references of Anaerolineaceae bacterium oral taxon 439 (Figure 4A; Table S7A). PAL006C was mapped against Abot439, Asot414, and Osot807 (Tables S7A–S7C; Figures S3–S5).

We were able to retrieve 192,093 reads unique to Abot439 from PAL005E, with 2.87X of average coverage, and 161,658 reads unique to Abot439 from PAL006C, with 2.63X of average coverage. Both samples displayed C to T terminal substitutions, characteristic of aDNA molecules (Table S7A; Figure S3). Phylogenetic analysis further supports the authenticity of the Abot439 sequences found in the concreted samples. A maximum likelihood tree made with 16,469 SNPs with a depth ≥3X found across 5 Titolo samples (2 from concreted tissue and 3 from archaeological calculus) and 34 published strains reveals close affinity with other Neolithic and Bronze age strains of the bacterium (Figures 4B, S6; Table S8). In particular, the Titolo strains cluster by the individual they were extracted from, regardless of the source from which the DNA was extracted. Nonetheless, node support is low for all Neolithic Italian trains of Abot439, indicating that given the quality of the retrieved genomes, there is not enough resolution to adequately distinguish between these genetically similar strains.

### Preservation of human and bacterial peptides

All extractions were processed in tandem for ancient peptide data (see STAR Methods). RAW files were searched against SwissProt, eHOMD, and the cRAP database with both pFind and novor.cloud.^36^ Both tools shared a 99.8% species identification rate, with only *Xanthomonas campestris* and *Xanthomonas euvesicatoria* (Two bacteria that infect plants) being uniquely identified by pFind (Figure S9). After filtration, 30,547 peptides, resulting in 6,457 proteins, were retrieved from all samples (Table S9).

**Figure 5:**
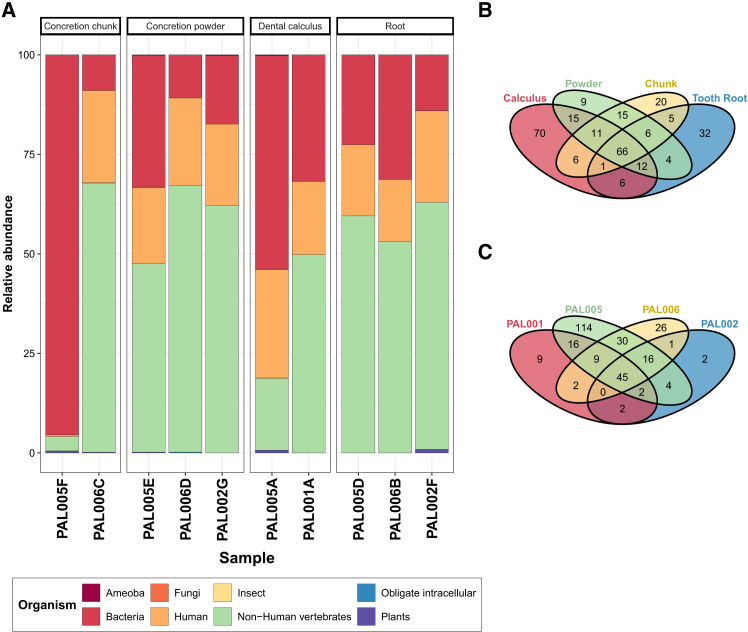
Relative proportions of peptides by organism and Venn diagrams showing number of species per sample type and individual (A) Stacked bar plot showing the relative abundance for peptide origin based on pFind output. (B) Venn diagram of species identified by pFind, categorized by sample type. (C) Venn diagram of species identified by pFind, categorized by individual.

Using pFind, we identified 13,657 unique peptides corresponding to 2,938 proteins. NQ deamidation rates for all peptides ranged from 3% to 40%, (Figure S7A), while average peptide lengths ranged from 14 to 24 amino acids (Figure S9). Peptides from *Homo sapiens* constitute about 20% of the identified peptides in all samples (Table S10), except for PAL005F (chunk of concretion) which contains only 0.4% of human proteins. The calculus sample PAL005A has the highest number of human peptides (*n* = 461), followed by concretions, which also contain a significant amount (Average = 234). Teeth have the lowest average number of human peptides (Average = 150). Although collagen is the most abundant peptide, the calculus samples (PAL001A and PAL005A) and concretions (PAL005E and PAL006C) samples also contain proteins associated with immune defense and components of the host’s blood, such as IgG and prothrombin (Table S10). Non-human vertebrate collagen comprises the largest part of the peptides analyzed (32%–68%), followed by bacterial peptides (9%–33%). However, samples PAL005F (concretion chunk) and PAL005A (calculus) exhibit a unique peptide distribution pattern, consisting of 95% and 53% of bacterial peptides, respectively (Figure 5A).

Venn diagrams were used to illustrate any potential protein species specific to different sample types or individuals (Figures 5B and 5C). We identified 45 protein species common to all individuals (Figure 5C), with 16 of these associated with the human oral microbes, 24 to non-human vertebrates, 4 to other bacteria and one to *Homo sapiens*. Of four individuals from Titolo, only PAL005 appears to have a unique protein community. In fact, 114 bacterial species were identified as unique to this individual, with very few peptides being poorly identified. The remaining peptides were assigned to parasites, non-human vertebrates, insects, or plants. Individual PAL005 hosts the highest number of HOM-specific species, with 88 bacterial species identified, followed by individuals PAL006 (*n* = 31) and PAL001 (*n* = 23).

Moreover, we identified HOM peptides specific to each sample type. Within these, 66 species are shared across all sample types, including *Homo sapiens*, 26 non-human vertebrates, and 39 bacteria, of which 35 are associated with the human oral microbiome. As expected, calculus contains the highest number of bacteria from the human oral microbiome (*n* = 90), followed by teeth (*n* = 54), chunk of concretions (*n* = 50) and concretions powder (*n* = 43) (Figure 5B).

Finally, to confirm the specificity of all the HOM candidate bacteria identified, we merged peptides whenever possible and performed a BLAST search against the NCBI nr database. We found specific peptides associated with HOM bacteria, such as *Actinomyces dentalis*, *Actinomyces* sp.,^37^ *Mobiluncus* sp.,^38^ *Arachnia propionica*,^39^ *Ottowia* sp.,^40^ *Cupriavidus* sp.,^41^ *Ralstonia pickettii*,^42^ *Prevotella*^43^ and *Capnocytophaga* sp.^44^ (Table S11).

Among the samples, one peptide was found in the teeth, 9 in the calculus, one in the concretions powder, and one in the concretions chunk. The proportion of specific peptides is not representative of the number of putative species found in each sample, as the teeth ultimately yielded the most specific peptides, rather than the calculus. Nevertheless, the ion coverage of these specific peptides remains relatively low. However, when considering the large number of peptides linked to these bacteria, it suggests that recovering human-associated bacteria, along with human-specific peptides, from bone-adhered concretions may be possible.

Interestingly, the metagenomic screening also confirms the presence of specific bacteria, whose peptides were identified using pFind and validated through BLAST (Table S11) in two dental calculus and one concretion sample. Notably, we detected reads associated with *Ralstonia pickettii* in PAL005F.

Furthermore, we evaluated the free online tool novor.cloud as a comparative approach for analyzing ancient peptides in the same set of samples. Unlike pFind, novor.cloud features a simplified, user-friendly interface that is freely accessible online, requires no installation, and supports fast cloud-based processing (300 MS/MS per second) on any standard laptop, regardless of the operating system. Aside from some distinctions, the distribution of peptides seems largely consistent between novor.cloud and pFind. The corresponding results and observations can be observed in Figures S7–S10 and in Tables S9 and S11.

## Discussion

aDNA preservation is significantly influenced by environmental factors such as temperature, pH, and moisture levels. Cold, dry, and stable environments are typically more conducive to DNA preservation.^45^ In skeletal remains, it is thought that DNA binds to the hydroxyapatite crystals in the bone matrix.^25^ The porous nature of bone facilitates the entrapment of cellular material, where DNA can become integrated within the crystal lattice or in microscopic voids.^46^ Teeth provide another robust source of aDNA, as the dentine and cementum are less prone to diagenetic changes than bone, often leading to better DNA preservation. These structures can safeguard DNA from external contaminants and physical degradation over extended periods.^12^ In sediments, DNA can adsorb to minerals through electrostatic interactions. Minerals like clay and silica are particularly effective at binding DNA molecules, which helps in their preservation; acidic conditions can enhance the binding affinity of DNA to minerals, thereby stabilizing it against degradation.^47^

In this study, we evaluated the preservation of ancient biomolecules in a previously overlooked material: sediment concretions tightly adhered to archaeological human remains. This contributes to ongoing studies into biomolecular preservation in alternative materials, developing our understanding of why and how human aDNA, microbial aDNA, and proteins bind to diverse substrates. The study focused on the Neolithic burial site of Titolo, located near the modern Bari, in Puglia (Southern Italy). The climatic data from Puglia region, show that the average annual temperature is 16.3 °C, with the warmest month being August (25.4 °C) and the coldest January (9.4 °C); the average annual rainfall is approximately 600 mm.^28^^,^^48^ During the Neolithic, fluctuations in climate, characterized by alternating arid and wet periods,^48^ may have influenced biomolecule preservation by affecting environmental conditions, such as temperature, moisture, and soil composition.

Results of our analysis show that the concreted sediment attached to the remains seems to be comparable to the underlying dental tissue. However, in comparison to their non-concreted counterparts, concreted tissues display poor human aDNA conservation, even in the case of tooth roots. Critically, the source material did not reach the minimum amount of unique human reads to perform population genetic analyses. This is a remarkable finding which should be considered in future biomolecular studies, and we caution that the utility of human remains covered in concretions may be limited. Nevertheless, it is possible that further studies of concretions surrounding human skeletal or dental remains, from other archaeological periods or environments, could yield different results. It is important to remark that we used a screening sequencing depth for the analysis, and thus, higher quality human data could be achieved either with deeper sequencing or capture.

In contrast to human aDNA content, retrieval of high-quality ancient microbial genomes is achievable using concreted tissue. Concretions likely form over skeletal and dental remains by persistent waterlogging of buried deposits and/or cycles of inundation and aridity. These environments probably create conditions in which endogenous human DNA degrades faster. On the other hand, bacterial cells, particularly gram-positive bacteria, often preserve better than eukaryotic (host) cells due to the presence of robust cell walls composed of thick peptidoglycan layers that offer additional protection against physical and chemical degradation.^49^ Indeed, Abot439, Asot414 and Osot807, retrieved from the layer of concretions covering the skeletal tissues, are gram-positive bacteria, thus it could be possible that DNA preserves as is more protected due to their cellular structure. Overall, the quality of the oral microbiomes retrieved from two of the concreted tissues (PAL005E and PAL006C) is high and comparable with that retrieved from the newly reported ancient dental calculus, as well as other published ancient and modern dental calculus. This is exemplified by the fact we manage to reconstruct two complete Anaerolinaceae bacterium oral taxon 439 with an average depth of coverage above 2.5X. The quality of the sequence was high enough to perform phylogenetic analysis with the strains. Phylogenetic placement of these demonstrates their affinity with other contemporary Italian strains,^32^ falling in a well-defined clade containing Bronze age and Neolithic strains, well differentiated from earlier Mesolithic strains or the modern diversity.^34^ Although they cluster together by individual extraction on the tree, exact placement within strains from the same site, or even other Neolithic samples, is difficult to assess due to insufficient resolution of the retrieved genomes and subsequent low support in the phylogeny. Although dental calculus is still the best source for investigating ancient oral communities, concreted elements as well as concreted sediments could still be still used to recover human oral species when other human remains are not available.

Due to proteins preserving better than DNA over time,^50^ we conducted palaeoproteomics analyses to determine whether concretions are suitable sources for ancient peptide analysis. We compared two tools using distinct peptide identification algorithms, pFind and novor.cloud. Our findings offer valuable insights into the composition and preservation of ancient peptides across different sample sources.

Human peptides were identified, as expected from previous studies,^12^^,^^51^ in tooth roots and dental calculus. For the first time, however, they have been successfully retrieved from archaeological sediment concretions. Although most of the recovered peptides are associated with collagen alpha-1(I) chain, collagen alpha-2(I) chain, or collagen alpha-2(V) chain, some samples, including dental calculus and concretions, contain human immunity- and blood-related peptides such as IgG and prothrombin^12^^,^^52^^,^^53^) (Table S10). These findings suggest that concretions could be a valuable source of host-derived proteins, potentially contributing to paleopathological and paleoserological studies for the indirect diagnosis of infectious diseases.^53^^,^^54^

Additionally, putative bacterial peptides associated with the human oral microbiome were identified in all samples, including concretions. Surprisingly, concretions contained nearly as many bacterial peptides as human teeth. Peptide blast analysis revealed the presence of distinct bacterial species, such as *Cupriavidus* sp/*Ralstonia pickettii* and *Prevotella* sp in concretion chunk and powder, respectively. However, bacteria found in calculus, such as *Mobiluncus* sp, and in chunk concretions, such as *R.pickettii*, while associated with the human oral microbiome, can also be present in soil,^55^ water,^56^ or the human vagina.^57^ Metagenomics and palaeoproteomics analyses of some samples revealed both ancient DNA and proteins from the same oral microbiome species, highlighting the advantages of combining these approaches for a more comprehensive understanding of substrates (Table S11).

Our study indicates that bacterial and human peptides are not uniformly preserved across different sample types, further supporting the hypothesis that certain microenvironments within calculus, teeth, and concretions may favor distinct preservation patterns. For instance, differences in mineralization, microbial activity, or exposure to environmental conditions may influence protein stability and degradation. In individual PAL005, certain bacteria are specific to calculus, teeth, or concretions. Overall, our results highlight the potential role of sediment concretions adhered to archaeological remains as both a direct and complementary source of ancient proteins from the host and its oral microbiome.

In this study, we investigated which biomolecules preserve in sediment concretions covering human remains. Our results show that in the absence of ancient dental calculus, or in studies that investigate the preservation of certain species in particular environments, this substrate can be a source of microbial reads and may be utilized to study ancient microbial genomes and proteins. Future research involving larger sample sizes is necessary to validate or challenge our initial findings. It is important to remark that the preservation of biomolecules is highly influenced by the storage conditions after excavation. Changes in temperature, humidity, together with other factors influence damage rates. Different studies have shown how post-excavation history of one sample can impact DNA preservation.^58^^,^^59^^,^^60^

Further studies, including geochemical methods and, possibly, experiment designs involving freshly excavated remains, could help in the investigation of such peculiar substrates, specifically to understand the conditions in which layers of concretion form over skeletal tissues, their content, and why certain biomolecules preserve better than others.

### Limitations of the study

Given the destructive nature of this exploratory analysis, the number of concretion-covered samples is low, pertaining to only 3 individuals from the same archaeological site. When sampling, we were careful to ensure that the original skeletal material or calculus was not sampled, and that only the layer of concretion was removed, but we cannot rule out the possibility that some small amount may have physically transferred. Furthermore, no chemical or geochemical analyses were carried out on the samples.

## Resource availability

### Lead contact

Further information and requests for resources and reagents should be directed to and will be fulfilled by the lead contact: Biancamaria Bonucci (biancamaria.bonucci@ut.ee).

### Materials availability

This study did not generate new unique reagents.

### Data and code availability

• The metagenomic shotgun sequencing data have been deposited at ENA: PRJEB80740. The mass spectrometry proteomics data have been deposited to the ProteomeXchange Consortium via the PRIDE^61^ partner repository with the dataset identifier PXD057010. Accession numbers are listed in the key resources table.
• The original code used to create a multifasta file containing variant positions found in the different strains can be found at https://github.com/tonidedios94/Utilities/blob/main/vcfFFCv.0.1.2.py.
• Any additional information needed to reanalyze the data in this paper is available from the lead contact upon request.

## Acknowledgments

We thank the Soprintendenza Archeologia, Belle Arti e Paesaggio (SABAP) per la Città Metropolitana di Bari for permission to analyze the Titolo remains, and SABAP per le Province di Barletta-Andria-Trani e Foggia for permission to analyze the Grotta Scaloria remains. A considerable part of this work was written at writing retreats and writing days organized by the Institute of Genomics, University of Tartu. The authors want to thank all the ANCESTORS team for discussions and comments. Data analyses were carried out with the facilities of the High-Performance Computing Center of the University of Tartu. We thank Tuuli Reisberg for assistance in data sharing and management. We thank the support of Sergo Kasvandik and the University of Tartu Proteomics Core Facility. The graphical abstract was created in BioRender (Bonucci, B. (2025) https://BioRender.com/nr4hk5b). This work is supported by the European Research Council Advanced Grant “Making Ancestors: The Politics of Death in European Prehistory” (no. 885137) (J.R., C.L.S., B.B., T.d.-D., J.E.T., S.P., and M.A.T); the Estonian Research Council grant PUT (PRG243) (A.S., C.L.S); and the European Union through the European Regional Development fund (project no. 2014–2020.4.01.16–0030) (C.L.S.).

## Author contributions

Conceptualization: C.L.S. and B.B.; data curation: B.B., T.d.-D., and J.E.T.; formal analysis: B.B., T.d.-D., and R.B.; funding acquisition: J.R., C.L.S., and M.A.T.; investigation: B.B., C.L.S., H.K., A.S., and J.E.T.; methodology: B.B., T.d.-D., R.B., and C.L.S.; project administration: C.L.S., J.R., and M.A.T.; resources: J.R., J.E.T., M.A.T., S.S., and F.R.; supervision: C.L.S. and J.R.; visualization: B.B., T.d.-D., R.B., and S.P.; writing – original draft: B.B. and C.L.S.; writing – review and editing: all authors.

## Declaration of interests

The authors declare no competing interests.

## STAR★Methods

### Key resources table

**Table:** 

REAGENT or RESOURCE	SOURCE	IDENTIFIER
**Biological samples**		

LLI1 calculus	This paper	PAL001A
LLI1 tooth root	This paper	PAL001AR
LLI2 calculus	This paper	PAL001B
LLC calculus	This paper	PAL001C
LRI2 calculus	This paper	PAL001DR
LRI2 tooth root	This paper	PAL001E
LLM3 tooth root	This paper	PAL001G
LLM2 tooth root	This paper	PAL002A
ULM2 tooth root	This paper	PAL002B
ULM2 buccal calculus	This paper	PAL002C
URC calculus	This paper	PAL002D
LRI2 calculus	This paper	PAL002DR
LRI2 tooth root	This paper	PAL002F
URP2 tooth root	This paper	PAL002G
Sediment	This paper	PAL003A
ULC calculus	This paper	PAL005A
ULC calculus	This paper	PAL005B
URM2 calculus	This paper	PAL005D
URI2 tooth root	This paper	PAL005E
Sediment	This paper	PAL005F
Sediment	This paper	PAL006B
LRI2 tooth root	This paper	PAL006C
Sediment	This paper	PAL006D
Sediment	This paper	SCA001
Femur	This paper	SCA002
Femur	This paper	SCA003
Femur	This paper	SCA004
Femur	This paper	SCA005
Femur	This paper	SCA006
Femur	This paper	SCA007
Femur	This paper	SCA008
Femur	This paper	SCA009
Femur	This paper	SCA010
Femur	This paper	SCA011
Femur	This paper	SCA012
Femur	This paper	SCA013
Femur	This paper	SCA014
Femur	This paper	SCA015
Femur	This paper	SCA016
Femur	This paper	SCA017
Femur	This paper	SCA018
Femur	This paper	SCA019
Femur	This paper	SCA020
Femur	This paper	SCA021
Femur	This paper	SCA022
Femur	This paper	SCA023
Femur	This paper	SCA024
Femur	This paper	SCA026
Femur	This paper	SCA027
Femur	This paper	SCA028
Femur	This paper	SCA029
Femur	This paper	SCA030
Femur	This paper	SCA031
Femur	This paper	SCA032
Femur	This paper	SCA033
Femur	This paper	SCA034
Femur	This paper	SCA035
Femur	This paper	SCA036
Femur	This paper	SCA037
Femur	This paper	SCA038
Femur	This paper	SCA039

**Chemicals, peptides, and recombinant proteins**		

Sodium Hypochlorite solution (13–15%)	N/A	CAS:7681-52-9
0.5 M EDTA pH 8.0	Fisher Scientific	Cat# BP24821
Ethanol 96%	Chemlab	Cat# CL00.0507.1000
dNTP Mix (25 mM each)	Thermo Fisher Scientific	Cat# R1122
dNTP Mix (10 mM each)	Thermo Fisher Scientific	Cat# R0192
BSA	Thermo Fisher Scientific	Cat# B14
HGS Diamond Taq	Eurogentec	Cat# TAQ-I011-5000+

**Critical commercial assays**		

MinElute PCR Purification Kit	QIAGEN	Cat# 28006
High Pure Viral Nucleic Acid LargeVolume Kit	Roche	Cat# 5114403001
NEBNext® End Repair Module	New England Biolabs	Cat# E6050L
NEBNext® Quick Ligation Module	New England Biolabs	Cat# E6056L
Bst DNA Polymerase, Large Fragment	New England Biolabs	Cat# M0275L
Qubit dsDNA HS Assay Kit	Thermo Fisher Scientific	Cat# Q32854
D1000 ScreenTape	Agilent	Cat# 5067-5582
D1000 Reagents	Agilent	Cat# 5067-5583
High Sensitivity D1000 ScreenTape	Agilent	Cat# 5067-5584
High Sensitivity D1000 Reagents	Agilent	Cat# 5067-5585

**Deposited data**		

Sequencing data	This paper	PRJEB80740
Protein data	This paper	PXD057010
Anaerolineaceae bacterium oral taxon 439 assembly ASM171754v1	The Forsyth Institute	https://www.ncbi.nlm.nih.gov/datasets/genome/GCA_001717545.1/
Mesolithic Anaerolineaceae bacterium oral taxon 439	Ottoni et al.^34^	PRJEB44313
Mesolithic Anaerolineaceae bacterium oral taxon 439	Fellow Yates et al.^33^	PRJEB34569
Modern Anaerolineaceae bacterium oral taxon 439	Velsko et al.^62^	PRJEB31185
18th century Anaerolineaceae bacterium oral taxon 439	Fellow Yates et al.^33^	PRJEB34569
Neolithic Anaerolineaceae bacterium oral taxon 439	Quagliariello et al.^32^	PRJNA791766
950-1200 AD Anaerolineaceae bacterium oral taxon 439	Warinner et al.^12^	SRP029257
Neanderthal Anaerolineaceae bacterium oral taxon 439	Weyrich et al.^13^	PRJNA685265
Actinomyces sp. oral taxon 414 assembly ASM127884v1	The Forsyth Institute	https://www.ncbi.nlm.nih.gov/datasets/genome/GCF_001278845.1/
Olsenella sp. oral taxon 807 assembly ASM118951v2	The Forsyth Institute	https://www.ncbi.nlm.nih.gov/datasets/genome/GCF_001189515.2/
Modern Human Calculus Metagenomes	Velsko et al.^62^	PRJEB31185
Modern Human Plaque Metagenomes	Human Microbiome project Consortium^63^	PRJNA48479
Modern Rural Human Gut Metagenomes	Rampelli et al.^64^	PRJNA268964
Modern Urban Human Gut Metagenomes	Human Microbiome project Consortium^63^	PRJNA48479
Modern Human Skin Metagenome	Oh et al.^65^	PRJNA46333
Modern Soil Metagenome	Bissett et al.^66^	PRJEB7626

**Oligonucleotides**		

NEBNext® Multiplex Oligos for Illumina® (Dual Index Primers Set 1)		Cat# E7600S
NEBNext® Multiplex Oligos for Illumina® (Dual Index Primers Set 2)		Cat# E7780S
IS1_adapter.P5 A∗C∗A∗C∗TCTTTCCCTACACG ACGCTCTTCCG∗A∗T∗C∗T	Meyer and Kircher^67^	Eurofins
IS2_adapter.P7 G∗T∗G∗A∗CTGGAGTTCAGACGTG TGCTCTTCCG∗A∗T∗C∗T	Meyer and Kircher^67^	Eurofins
IS3_adapter.P5+P7 A∗G∗A∗T∗CG GAA∗G∗A∗G∗C	Meyer and Kircher^67^	Eurofins

**Software and algorithms**		

QGIS 3.38.1		https://qgis.org/
SAMtools v.1.12	Danecek et al.^68^	http://www.htslib.org/
AdapterRemoval2 v.2.3.3	Schubert et al.^69^	https://github.com/MikkelSchubert/adapterremoval
prinseq v.0.20.04	Schmieder and Edwards^70^	https://prinseq.sourceforge.net/index.html
bbmap v.38.18	Bushnell^71^	https://github.com/BioInfoTools/BBMap
BWA v.0.7.17-r1188	Li and Durbin,^72^ Li^73^	https://github.com/lh3/bwa
Picard v.2.20.8	Broad Institute^74^	https://broadinstitute.github.io/picard/
KrakenUniq v.1.0.4	Breitwieser et al.^30^	https://github.com/fbreitwieser/krakenuniq/tree/master
SourceTracker2 v.2.0.1.dev0	McGhee et al.^31^	https://github.com/caporaso-lab/sourcetracker2/tree/master
MapDamage2 v.2.2.1	Jónsson et al.^75^	https://ginolhac.github.io/mapDamage/
Qualimap2 v.2.2.2-dev	Okonechnikov et al.^76^	http://qualimap.conesalab.org/
compositions v.2.0–8	Van der Boogart^77^	https://cran.r-project.org/web/packages/compositions/index.html
mixOmics v.6.23.4	Rohart et al.^78^	https://bioconductor.org/packages/release/bioc/html/mixOmics.html
GATK v.3.7	McKenna et al.^79^	https://gatk.broadinstitute.org/hc/en-us
RAxML v.8.2.12	Stamatakis^80^	https://bioweb.pasteur.fr/packages/pack@RAxML@8.2.12
bcftools v.1.8	Danecek et al.^68^	https://samtools.github.io/bcftools/howtos/publications.html
pFind v.3.2.0	Chi et al.,^81^ Shao et al.^82^	https://github.com/pFindStudio/pFind3
BlastP	Altschul et al.^83^	https://blast.ncbi.nlm.nih.gov/Blast.cgi?PAGE=Proteins
Megan v.6.25.1	Huson et al.^84^	https://software-ab.cs.uni-tuebingen.de/download/megan6/welcome.html
Venny v.2.0.2	Oliveros^85^	https://bioinfogp.cnb.csic.es/tools/venny/index2.0.2.html
bedtools v2.30.0		https://bedtools.readthedocs.io/en/latest/
circlize v.0.4.16	Gu et al.^86^	https://github.com/jokergoo/circlize
R v6_2.5.1		https://www.R-project.org/
ggtree v.3.8.2	Yu et al.^87^	https://www.bioconductor.org/packages/release/bioc/html/ggtree.html
vcfFFC v.0.1.2.py	This paper	https://github.com/tonidedios94/Utilities/blob/main/vcfFFCv.0.1.2.py

### Experimental model and study participant details

The DNA was extracted from 62 skeletal samples in 62 extracts, 62 double-stranded, dual-indexed libraries were generated. More detailed information about the archaeological sites of this study is given below.

All necessary permits were obtained for the described study, which complied with all relevant regulations. The *Soprintendenza Archeologia, Belle Arti e Paesaggio per le province di Barletta-Andria-Trani e Foggia* authorised this research on the human remains from Grotta Scaloria (stored at the Department of Archaeology, Cambridge, UK). The human remains from Titolo are on loan to the *Museo Giuseppe Sergi* (Sapienza University, Rome) and their analysis was approved by the *Soprintendenza Archeologia, Belle Arti e Paesaggio per la Città Metropolitana di Bari*.

#### The archaeological background of the site of Titolo

##### Biancamaria Bonucci, Jess E. Thompson & Sofia Panella

Titolo is located within the modern town of Palese-Macchie (Bari, Puglia, Italy). The human remains included in this study were excavated between 2012 and 2014 by the *Soprintendenza per i Beni Archeologici della Puglia* (Sivilli S. 2018, unpublished). Four excavation campaigns uncovered a large area of Neolithic occupation, including a funerary space at the SE margin of the settlement, which was bordered by a large drystone wall. The archaeological deposits were a mere 1–1.5 m deep, resting on a sterile layer of *terra rossa* soils just above the limestone bedrock (Sivilli S. 2018, unpublished). The sediment mostly comprised soft fine acidic sands and silty-sands of gray to yellowish-brown color; occasionally, darker grey or black ashy deposits were encountered (Sivilli S. 2018, unpublished).

Occupation at Titolo spanned four main phases throughout the Neolithic, identified on the basis of ceramic styles: *impresa evoluta* (late early Neolithic)*, bande rosse, Serra d’Alto* (both Middle Neolithic)*,* and *Diana* (late Neolithic). In the funerary area, ten features containing human remains (T1–T10) were identified: nine of these comprised shallow grave pits containing primary single burials, while T10 was a small circular pit containing selected disarticulated bones from three individuals. Of these twelve individuals, eight were adults and four were nonadults. Five radiocarbon dates from primary burials are available. Three radiocarbon dates have been previously published: LTL-19755A: 6815 ± 45BP (T9); LTL-15552A: 6541 ± 50BP (T2); LTL-14541A: 6230 ± 50BP (T6).^88^ We obtained two further radiocarbon dates, while the sample from T8 failed due to insufficient collagen preservation: SUERC-106332: 6452 ± 29BP (T3); SUERC-106949: 6381 ± 24BP (T7). On the basis of these dates, it is clear that T9 is the most ancient burial, T2, T3 and T7 were deposited in a similar timeframe around the mid-6th millennium BCE or in the following century, while T6 was probably buried in the final quarter of the 6th millennium BCE.

The graves were mostly shallow (c. 40 cm deep), circular, oval, or sub-oval in shape, sometimes with a concave base, and often partly or fully lined by small stones (Sivilli S. 2018, unpublished). Some were disturbed or intercut by other graves or by the stone floor or wall structures which had been frequently dismantled and rebuilt. Graves T1–T5 all exhibited evidence of inter-cutting and disturbance and, in general, the dense distribution of stones in and around the burial pits had a significant taphonomic impact on the preservation of the human remains. Grave T9 was connected to a floor containing traces of *concotto* (remains of a ceramic oven), as well as bordered by several older phase walls.

The skeletal remains from T1–T5 and T7 are all poorly represented, <50% complete and exhibit poor or fair preservation. Individuals T6, T8 and T9 are more complete (75–99%), with fair preservation of the bone noted where observable. Evidence of erosion to the bone cortices is minimal, but most extant remains from most individuals were partially or entirely obscured by sediment concretion. Concretions were homogeneous in texture and colour, consisting of a hard, dark grey sediment tightly adhered to the bone surfaces. These concretions had preserved the *in-situ* positions of bones within some graves, suggesting that some burials had decomposed in open spaces. It is probable that only thin soil coverings were placed over the bodies, mixed in with the stones included in the graves.^89^

Regarding peptide analysis of human oral microbiome (eHOMD) in the Titolo individuals, it is interesting to compare these results to macroscopic observations of dental health and pathology. However, it is important to note that the same concretions that form the subject of this study frequently also covered the alveolar margins and tooth crowns, preventing their observation. Dentitions were observable for nine of the twelve individuals (absent for T1, T4 and T10C). Carious lesions were present in three individuals (T2, T3, T6) on a total of eight teeth (prevalence 5.3% out of 151 total teeth; prevalence 8.6% out of 93 possible observations). On one tooth each from T2 (lower right third molar) and T3 (upper left second molar), 2 carious lesions were observed. Calculus was observed on 55 teeth (prevalence 36.4% out of 151 teeth; prevalence 50% out of 110 possible observations). Periodontal disease, where observable, is mild to moderate in the adult individuals; continuous eruption is also observed in much of the adult dentition, further indicated by the presence of carious lesions on the cementum-enamel junction (CEJ) in several individuals.

In T2, on the upper left third molar, a large carious lesion was observed on the mesial contact area of the crown and extended slightly onto the root; on the lower right third molar, caries was observed on the lingual and distal aspects at the CEJ. In T3, the upper left second molar displayed carious lesions at the CEJ on the mesial and distal aspects, and the upper left third molar presented a corresponding carious lesion on the mesial aspect of the CEJ. In T6, 4 teeth presented caries: the upper right first premolar presented a small cavity in the distal contact area; the upper left canine presented a small cavity in the distal contact area, the lower right first premolar presented a large cavity in the distal contact area and the adjacent second premolar presented a corresponding small cavity in the mesial contact area. Carious lesions on the root surface/CEJ are initiated when the root surface is exposed either due to periodontal disease or to compensatory eruption, usually during mid-to-late adulthood.^90^ In T2 and T3, it is the second and third molars which are affected, regions of the dental arcade in which it can be difficult to maintain oral hygiene, especially in the interproximal spaces. T6 displays a pattern of dental pathology consistent with increased age; in addition to carious lesions on one canine and 2 premolars, at least 3 teeth appear to have been lost ante-mortem (upper right second premolar, lower left first molar, lower right second molar), and 3 teeth are worn inferior to the CEJ (presenting as functional root stumps: two loose mandibular incisors, and the lower left second premolar, which has rotated mesially probably following the loss of the adjacent first molar). Further incidences of ante-mortem tooth loss are observed in T8 (lower left first and third molars), T9 (upper left third molar) and T10A (lower left first and third molars, lower right central incisor).

Calculus is observed on dentitions from T2, T3, T5, T6, T7, T8, and T9, although for most individuals, observations of calculus were obfuscated on several or most teeth. In many cases, it was observable as a small area of pinkish-beige calculus deposit underlying dark grey concretion. When concretion covered the calculus, the extent of calculus over the enamel surface and/or in the subgingival space could not be clearly determined. In T2, one maxillary and three mandibular incisors exhibited slight to considerable supragingival calculus deposits on the labial surfaces. In T3, 23 of 28 observable teeth presented at least slight supragingival calculus deposits on the labial or buccal surfaces, as well as slight calculus deposits on the lingual surfaces of 5 teeth; on the upper left first molar and lower lateral incisors, subgingival deposits were present. In T5, one of the two extant teeth (upper right first premolar) presented slight supragingival calculus on the buccal surface. In T6, 8 teeth presented slight to medium supragingival calculus on the labial or buccal surfaces. The young child in T7 presented slight supragingival calculus on the upper right second deciduous molar. In T8, 7 teeth exhibited slight to medium supragingival calculus on the labial or buccal surfaces. T9 presented slight to medium supragingival calculus on six teeth on the labial or buccal surfaces.

### Method details

All the laboratory work was performed in dedicated ancient DNA laboratories at the Estonian Biocentre, Institute of Genomics, University of Tartu, Tartu, Estonia. The library quantification and sequencing were performed at the Estonian Biocentre Core Laboratory. The main steps of the laboratory work are detailed below.

#### DNA extraction

Amounts of 20–250 mg of tooth roots, chunk from the concretion-covered tissues and powder of concretion were sampled. The drill bits and core drill were sterilised in between samples with 6% (w/v) bleach followed by distilled water and then ethanol rinse. Root portions of teeth were removed with a sterile drill wheel. Afterward, the root portions were weighed to calculate the accurate volume of EDTA (20x EDTA [μl] of sample mass [mg]). The calculated amount of EDTA was added to the root portions, while 500 μL of EDTA were added to the chunks and the powder. The tubes were incubated 24 h on a nutating mixer at 40°C.

Up to 10 mg of dental calculus were removed with a sterile dental scaler and weighed. Next, 500 μL of EDTA were added to the samples and the tubes were left rotating for 5–15 min at room temperature on a nutating mixer. Afterwards, the EDTA was removed and stored for later testing. 500 μL of fresh EDTA were added again to the dental calculus and the tubes were then incubated at room temperature on a nutating mixer for 72 h.

The DNA extracts of root portions were then concentrated using the Vivaspin Turbo 15 (Sartorius), and then with the other extracts, purified in large volume columns (High Pure Viral Nucleic Acid Large Volume Kit, Roche) using 10 x mL of PB buffer, 1 mL of PE buffer and 100 μL of EB buffer (MinElute PCR Purification Kit, QIAGEN) per sample. For the elution of the endogenous DNA, the silica columns were transferred to a collection tube to dry and followed in 1.5 mL DNA lo-bind tubes (Eppendorf) to elute. The samples were incubated with 100 μL EB buffer at 37°C for 10 min and centrifuged at 17,949 x *g* for 2 min. After centrifugation, the silica columns were removed, and the samples were stored at −20°C. Only one extraction was performed and 30 μL were used for libraries.

#### Library preparation

Sequencing libraries were built following established protocols.^67^^,^^91^ Three verification steps were implemented to make sure library preparation was successful and to measure the concentration of dsDNA/sequencing libraries - fluorometric quantitation (Qubit, Thermo Fisher Scientific), parallel capillary electrophoresis (Tape Station, Agilent Technologies) and qPCR (KAPA Library Quantification Kit (Illumina® platforms)).

#### DNA sequencing

DNA was sequenced using the Illumina NextSeq500/550 High-Output paired-end 150 cycle kit.

#### Processing of sequencing data

Before mapping, the raw sequences were trimmed with AdapterRemoval v.2.3.3.^69^ In order to avoid spurious matches in the following analysis, we discarded all sequences shorter than 30bp and with a quality below 20.

To further reduce the spurious assignment of generated sequences to evolutionary conserved regions, we removed low complexity sequences using prinseq v.0.20.4 and a dust value of 7.^70^ Finally, we collapsed all duplicated reads using bbmap v.38.18 tool suite.^71^

#### Mapping to the human genome and assessing the authenticity of ancient human sequences

Sequences were aligned to the human reference genome 37 using *bwa backtrack*, with edit distance and seeding modified to account for aDNA damage (-n 0.01 and -l 16000). Alignments were then converted, sorted, and indexed using samtools.^68^ Picard^74^’s MarkDuplicates module was used to remove duplicates. We then kept only sequences with a mapping quality equal or above 30. Mapping statistics were generated using Qualimap2^76^ and bedtools. As DNA degrades over time, real aDNA reads show signs of decay. Postmortem damage at the ends of the DNA sequences can result in cytosine (C) to thymine (T) and guanine (G) to adenine (A) nucleotide changes at the 5′ end and 3′ end, respectively^46^^,^^92^. This phenomenon becomes more frequent toward the ends of the fragments.^93^^,^^94^ Thus, we used mapDamage2.2.1^75^ to quantify the post-mortem damage.

#### Genetic sex determination

Genetic sex was calculated using the script sexing.py from,^95^ which allows to estimate the number of reads mapping to chrY out of all reads mapping to either X or Y chromosome.

#### Microbial screening

After preprocessing, all the reads that didn’t map to the human genome were processed with KrakenUniq and the standard MicrobialDB.

#### Microbial SourceTracking analysis

We have filtered our raw KrakenUniq reports using the E-score derived from the proportion, in each taxonomic level, of kmers by read per genome coverage. Higher E-scores denote a higher distribution of the matching hits along the reference genome, and thus, the higher the probability that the hit is genuine. Data for Source Tracker analysis was curated as described in.^96^ We have considered valid microbial species with a minimum E-score of 7.^97^

To estimate the proportion of reads stemming from various microbiota, we have used SourceTracker2,^31^ a Bayesian source-prediction tool. The analysis with Sourcetracker was performed on normalised bacterial taxa reads abundance at the species level. We have used a custom metagenomic dataset of sources,^63^^,^^64^^,^^65^^,^^66^ including modern human dental calculus, human oral plaque, skin, soil, and gut from the European Nucleotide Archive (ENA), as sources as described in de-Dios and colleagues.^96^ We individually merged each filtered sample report to this reference dataset, keeping species with at least 200 reads in the Reference samples, and 50 reads in the target sample (to account for lower abundance of microbial reads in ancient samples), and discarding species with an abundance below 0.02% in the entire dataset. We then ran SourceTracker2 with our target sample as sink, using a refraction value of 100 for sinks and sources.

#### Clustering analysis

We have selected a comprehensible dataset of oral associated microbial species from the eHOMD and as inferred by SourceTracker2 (Calculus, Subgingival Plaque, Supragingival Plaque) (Table S12). We extracted those species from our samples’ raw KrakenUniq report. We also added 25 ancient published calculus, and 30 modern plaque and calculus samples.^32^^,^^34^^,^^62^^,^^98^ We filtered those resulting datasets for an E-score of 7, and a minimum number of reads of 10. After merging those, we have normalised each sample for library size, and we have discarded species with a representation below 0.02%. This resultant dataset was then renormalised using a *clr* transformation^77^ and a Principle Component Analysis (PCA) was computed using mixOmics R package.^78^ Resultant sample values and loadings were visualised using R package ggplot2.^99^

#### Microbial genome mapping and authentication

Sequencing data from three dental calculus samples and two test samples were aligned to the reference genome of the oral bacteria species of Anaerolineaceae bacterium oral taxon 439 (NZ_CP017039), Actinomyces sp. oral taxon 414 (NZ_CP012590) and Olsenella sp. oral taxon 807 (NZ_CP012069.2) (Tables S6A–S6C; Figures S3–S5) using *bwa backtrack* with an edit distance of 0.01 (-n 0.01), a gap open penalty of 2 (-o 2) and seeding disabled (-l 10,000).^72^^,^^73^ Alignments were then converted, sorted, and indexed using samtools.^68^ We used Picard’s MarkDuplicates module to remove all duplicates. Finally, we kept aligned sequences with a mapping quality equal or above 30. Basic mapping statistics were generated using Qualimap2^76^ and bedtools. For authentication, aDNA associated deamination profiles were computed using mapDamage2.2.1.^75^ Reference mappability was calculated as the fraction of covered regions given simulated reads of length 50 at a depth of 30. We displayed normalised genome coverage along the reference genome, mappability, GC content and gene content using the R package circlize.^86^

#### Anaerolineaceae bacterium oral taxon 439 phylogenetic analysis

We have selected a total of 34 samples originating from previous studies,^12^^,^^13^^,^^32^^,^^33^^,^^34^^,^^62^ and we mapped them against the Anaerolineaceae bacterium oral taxon 439 reference genome as described above. Those included a selection of Neanderthal, Mesolithic, Neolithic, Bronze Age, and Modern strains of the bacterium. To those, we added 5 of our newly generated samples with more than 3X of average genomic coverage. Those 3 calculus samples (PAL003A, PAL005A and PAL005B) and 2 concreted tissue samples (PAL005E and PAL006C). We have generated a wide genome variant calling using GATK v3.7 algorithm *UnifiedGenotyper,* with the option *--emit_all_sites*.^79^

To explore the phylogenetic relationship of the newly retrieved samples with other ancient and modern strains of the bacterium, we have created a SNP dataset. First, we merged all resultant VCFs using *bcftools merge* with the flag *–merge all*. We then used an in-house python script to create a multifasta file containing variant positions found in the different strains (available at https://github.com/tonidedios94/Utilities/vcfFFCv.0.1.2.py). We selected an heterozygous ratio of 0.9 to account for terminal aDNA associated C to T substitutions (-r 0.9), a minimum genotype quality of 30 to ensure the sufficient likelihood of the called position (-q 30), a minimum depth of 3 which is a standard minimum depth used in phylogenetic analysis of ancient samples(-d 3), that a SNP is present in at least 75% of the strains in the dataset (-m 0.75), and a minimum distance between positions of 3 bp (-p 3). The heterozygous ratio value will consider a position as homozygous for the majoritarian allele if the ratio of allele depth is above 90%, masking the position with an N if they do not reach this threshold. This was done to consider possible multiple strains and associated terminal aDNA damage. Additionally, all indels were discarded, since they are difficult to be called in low coverage ancient data using current existing algorithms. The resultant dataset comprised a total of 16,469 variant biallelic positions covered across the 39 samples (Table S8).

The resultant SNP dataset was used to create a maximum-likelihood (ML) phylogenetic tree. This was achieved using RAxML v8.2.12,^80^ using the *GTRGAMMA* model and 1,000 bootstraps, and rooted to the Neanderthal strain from *ElSidron1*^13^*.* We chose a 1,000 bootstrap as is the standard in ancient microbial phylogenies and similarly sized datasets. The inferred best tree and bootstrap values were visualised using ggtree.^87^

#### Proteomics analyses

##### Isolation of peptides

The pellets and 50 μL of supernatant were retained for proteomics. In order to perform protein extraction, 150 μL of 2M guanidine HCl were added, then 20 μL each of 100 mM CAA and TCEP. Samples were incubated at 99C for 10 min then allowed to cool. 10 μL of a mix of Sera Mag beads was added along with 230 μL of 100% Ethanol and incubated at 24°C for 5 min on a shaker. Beads were pelleted using a magnetic rack and the supernatant removed. Beads were washed three times with 80% ethanol then 150 μL of TEAB and 1 μL trypsin were added to each tube. After checking that the pH was between 7 and 9, the tubes were incubated for overnight digestion (12–18 h) at 37°C and light shaking was applied. In the morning, Pierce™ C18 Tips, 100 μL bed were used to immobilise the peptides. Tips were prepared with methanol, AT80 and 0.1% TFA. Samples were run through the tips twice and followed by two rounds of 150 μL 0.1% TFA. Tips were stored at −20°C until delivered to the Proteomics Core Facility at the University of Tartu.

##### Peptide sequencing

Samples were eluted from C18 StageTips and reconstituted in 21 μL of 0.5% TFA. LC-MS/MS analysis was carried out by loading the entire sample to a 0.3 × 5 mm trap-column (5 μm C18 particles, Dionex) using an Ultimate 3500 RSLCnano system (Dionex, California, USA). Peptides were eluted to an in-house packed (3 μm C18 particles, Dr Maisch, Ammerbuch, Germany) analytical 50 cm × 75 μm emitter-column (New Objective, Massachusetts, USA) and separated at 250 nL/min with an A to B 8–40% 1.5 h gradient (buffer A: 0.1% formic acid, buffer B: 80% acetonitrile +0.1% formic acid). Both the trap- and analytical columns were operated at 40^o^C. Separated peptides were on-line electrosprayed to a Q Exactive HF (Thermo Fisher Scientific) mass spectrometer via a nano-electrospray source (positive mode, spray voltage of 2.6 kV). The MS was operated with a top-12 data-dependent acquisition strategy. Briefly, one 350–1,400 m/z full MS scan at a resolution setting of R = 60,000 at 200 m/z was followed by higher-energy collisional dissociation fragmentation (normalised collision energy of 26) of the 12 most intense ions (z: +2 to +5) at R = 30,000. MS and MS/MS ion target values were 3,000,000 and 100,000 ions with 50 and 41 ms injection times, respectively. MS/MS isolation was carried out with 1.6 m/z isolation windows. Dynamic exclusion was limited to 35 s. Peptide match was set to „Preferred“ to select isotopic features reminiscent of peptides for data-dependent scanning.

##### Database search and result filtering

Raw mass spectrometry data were searched for each samples using novor.cloud^100^ and pFind (v.3.2.0)^81^^,^^82^ against all proteins in SwissProt (version dated 2 may 2024), cRAP (version dated of 4 march 2019) (https://www.thegpm.org/crap/) and eHOMD (version V3.1) databases. For both searches, fixed modification was set to include carbamidomethylation of cysteine and variable post-translational modifications (PTMs) were set to include proline hydroxylation, glutamine and asparagine deamidation, methionine oxidation, and pyroglutamate formation from glutamine and glutamic acid. Searches were conducted with trypsin full-specific digestion. Precursor mass tolerance was set to 15 ppm and fragment mass tolerance to 0.02 Da and the false-discovery rate of peptide spectrum matches equal ≤1.0%. pFind peptides were filtered based on their score. Only peptides with a score below or equal to 0.01 were considered. Protein matches were considered only if supported by a minimum of two unique peptide spectral matches (PSMs), either in novor.cloud or pFind. All contaminants from our samples were identified and removed (at the exception of human collagen) using the peptide identified within our extraction blank and the cRAP database.

##### Peptide analysis

For both novor.cloud and pFind, we measured the proportion of all identified post-translational modifications (PTMs) relative to the total number of amino acids. We quantified in particular glutamine/asparagine deamidation along with the peptide length distributions to assess the quality of preservation of our samples according to literature data on ancient peptides. Then, we classified our peptides by origin (human, vertebrate, bacteria, plants, fungi, obligate intracellular, amoeba, insect) based on top hit identification from novor.cloud and pFind. To examine and quantify variations in peptide identification between novor.cloud and pFind, we utilised Venny 2.0.2^85^ and R Studio (Version 2024.04.0 + 735) to generate Venn diagrams. The same method was used to assess the presence of specific peptides based on sample source and individual. Additionally, we compared bacterial peptides against an in-house database, including the Human Oral Microbiome Database (eHOMD) (version 3.1), the SMAG catalog,^101^ RefSoil,^102^ and the European Soil Diversity database, to classify bacterial peptides. Finally, All our no-PTMs bacterial peptides were blasted against the NCBI nr database using BLASTP,^83^ filter to keep only the peptides yielded 100% identity and coverage with reference sequences and then parsed using MEGAN.^84^

### Quantification and statistical analysis

All the figures presented in the manuscript were created using R (v2024.04.0 + 735) and domain-specific tools. The PCA was computed on clr-transformed microbial abundance data (at the species level) using the mixOmics R package. Source prediction was performed with SourceTracker2 using read counts at the species level with a rarefaction depth of 100. KrakenUniq reports were filtered for valid microbial species (E-score ≥7, species-level abundance ≥0.02%, minimum 200 reads in reference samples, 50 in the samples under study).

Genomic alignments were filtered using a mapping quality >30 and authenticity metrics (mapDamage2.2.1). SNP calling thresholds included: GQ ≥ 30, depth ≥3, minimum 0.9 allele ratio, SNP present in ≥75% of strains, 3 bp minimum spacing. Phylogenetic reconstruction used RAxML (GTRGAMMA, 1,000 bootstraps). No formal statistical significance testing was applied. The exact parameters are reported in the figure legends.

For proteomics data, peptides with a pFind score ≤0.01 and ≥2 PSMs were retained. Origin classification and comparative quantification were performed using R and Venny2.0.2.

## Supplemental information

Document S1. Methods S1, Figures S1–S10, Table S6, Supplementary bibliography

Table S1. Full sample information (A–C), related to Table 1

Table S2. Mapping Statistics Human DNA, related to Figure 2

Table S3. KrakenUniq raw reports (test set), related to STAR Methods

Table S4. KrakenUniq raw reports (control set), related to STAR Methods

Table S5. Sourcetracker2 results (A-Q), related to Figure 3

Table S7. Mapping statistics for the alignment of chosen samples to the reference genomes of three detected microbial species, related to Figure 4 and STAR Methods

Table S8. Phylogenetic dataset coverage, related to Figure 4

Table S9. Proteomics screening, related to Figure 5

Table S10. Human proteins identified with pFind, related to STAR Methods

Table S11. BLAST results for HOM bacteria identified with pFind, related to STAR Methods

Table S12. Taxa IDs of oral species selected for custom dataset, related to Figure 3
