# Supplementary material for: Ancient microbial DNA and proteins preserve in concretions covering human remains
Source: iScience. 2025 Jul 23;28(8):113182. doi: 10.1016/j.isci.2025.113182 (PMC12355549; doi:10.1016/j.isci.2025.113182)
Supplement: Document S1. Methods S1, Figures S1–S10, Table S6, Supplementary bibliography [file mmc1.pdf]

iScience, Volume *28*

Supplemental information

Ancient microbial DNA and proteins preserve in concretions covering human remains

Biancamaria Bonucci, Toni de-Dios, Rémi Barbieri, Jess Emma Thompson, Sofia Panella, Francesca Radina, Sandra Sivilli, Helja Kabral, Anu Solnik, Mary Anne Tafuri, John Robb, and Christiana Lyn Scheib

Methods S1: Archaeological context of Grotta Scaloria

John E. Robb & Jess E. Thompson

Grotta Scaloria is a large cave located in the modern town of Manfredonia (Foggia, Puglia, Italy), which was formed by karstic activity at the interface between terrace deposits in the Gargano promontory. The cave was originally discovered in 1931 and has been excavated episodically since the first excavations in the Upper Chamber between 1931–1936 led by Quintino Quagliati[1]. The Lower Chamber was discovered by speleologists during the 1960s, leading to a period of systematic excavations led by Santo Tinè and Marija Gimbutas[2–4]. Apart from early, sporadic use in the Late Upper Palaeolithic, the main frequentation of the cave commenced in the early 6th millennium BCE, with a period of intense activity and funerary use between 5500–5200 cal BCE during the middle Neolithic, and occasional use in the Late Neolithic[5].

The Upper Chamber was regularly used for both settlement and funerary purposes. Alongside habitation evidence, including animal keeping and food preparation, there is evidence for the secondary deposition of remains (some of which were defleshed) and occasional primary burial[6,7]. The human remains included in this study pertain to samples from long bones in disarticulated deposits from Trenches 4, 6, 8 and 10 and Area A. The Lower Chamber appears to have been reserved for specific rituals which were carried out only during a short period in the Middle Neolithic. The Lower Chamber is associated with the so-called 'cult of the waters': an artificial pool has been cut into the rock and large ceramic vessels were positioned underneath dripping stalactites[8]. Only sporadic human remains were found in the Lower Chamber, consisting of one skeleton and one mandible.

A large assemblage of human remains has been excavated from Grotta Scaloria across all excavation campaigns, most of which have now been united to form an integrated study. On

the basis of the excavated remains, at least 31 individuals (19 adults and 12 nonadults) are represented[6,7], although the cave is not fully excavated. While primary burials were found in the earlier excavations, later campaigns predominantly identified secondary deposits of disarticulated bones, often in groups or clusters which contained the incomplete remains of multiple individuals of different ages[7].

Supplementary figures

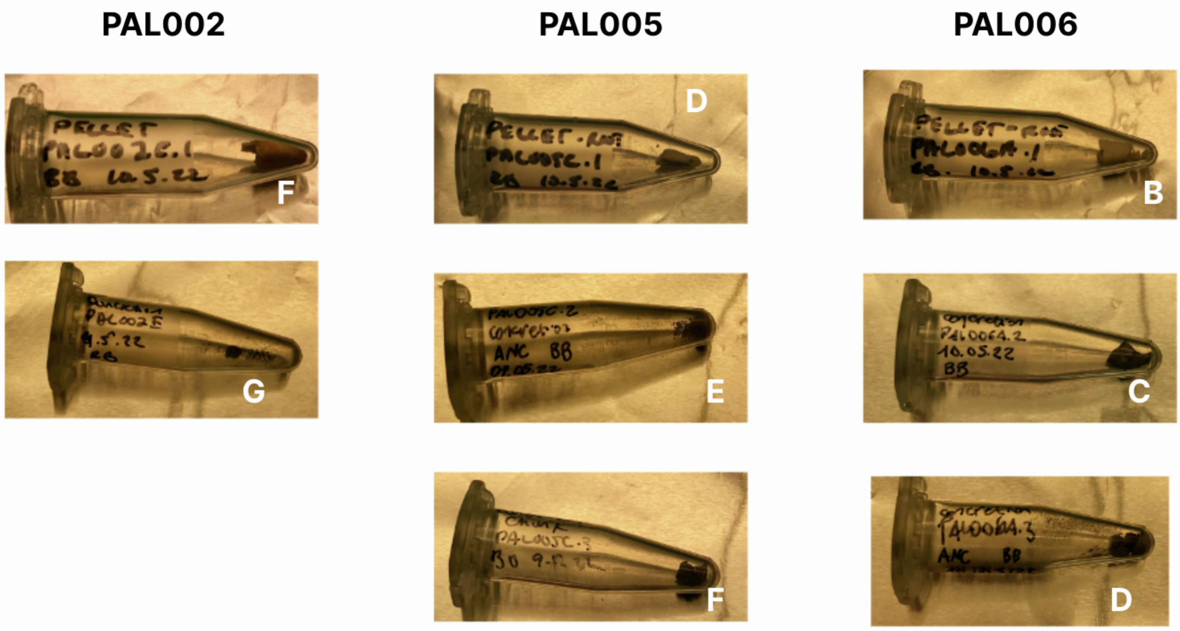

Figure S1. Photos of samples inside the lab. Tubes showing the samples taken, related to Figure 1.

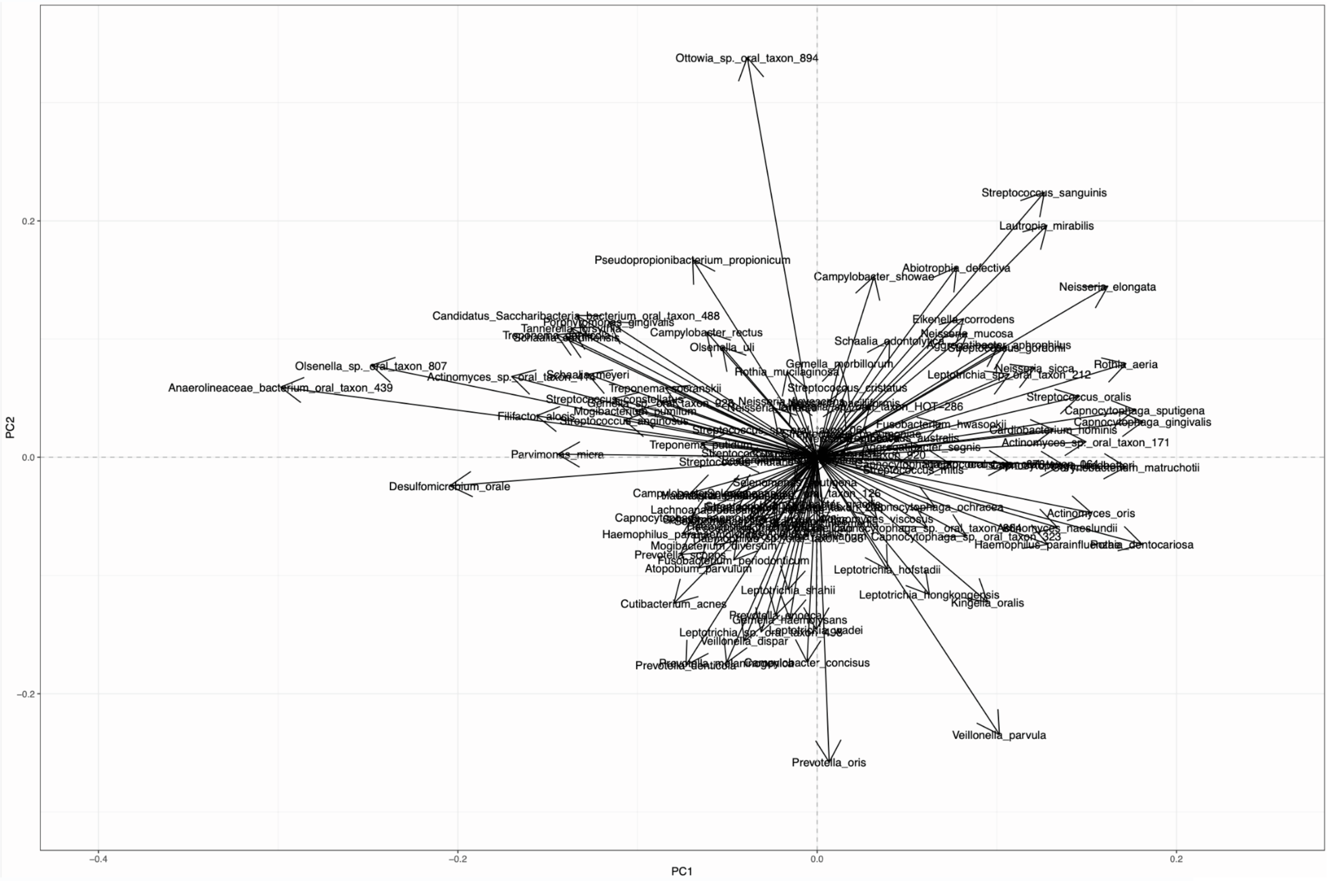

Figure S2. PCA loadings for species characterized as oral. Related to Figure 3.

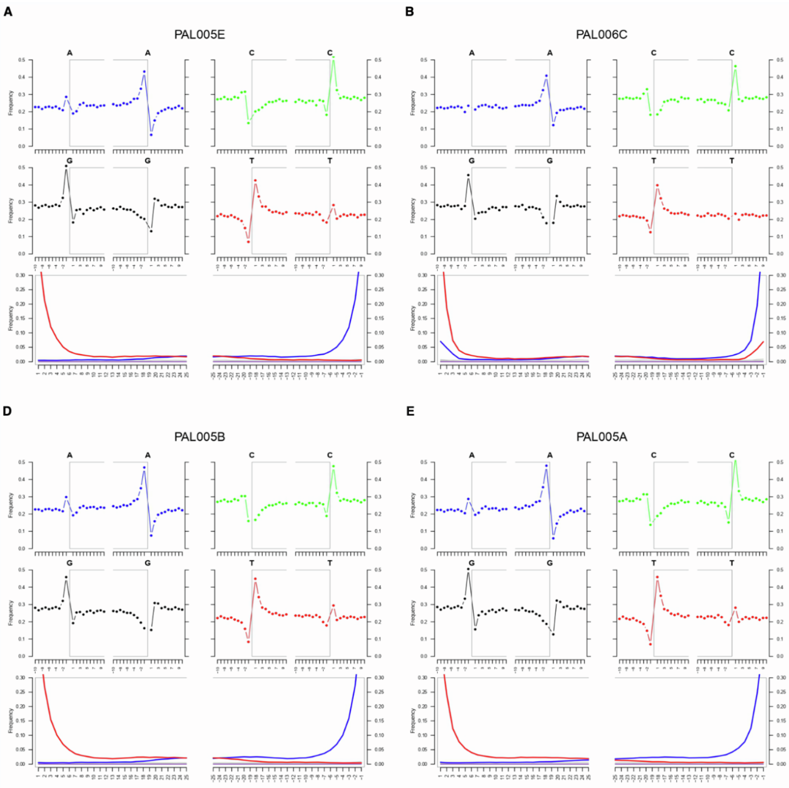

Figure S3. Mis-incorporation plots produced by mapDamage 2.0 after alignment of target samples to Anaerolinaceae bacterium oral taxon 439 reference genome. A) PAL005E. B) PAL006C. C) PAL003A. D) PAL005B. E) PAL005A. Related to Figure 4 and STAR Methods.

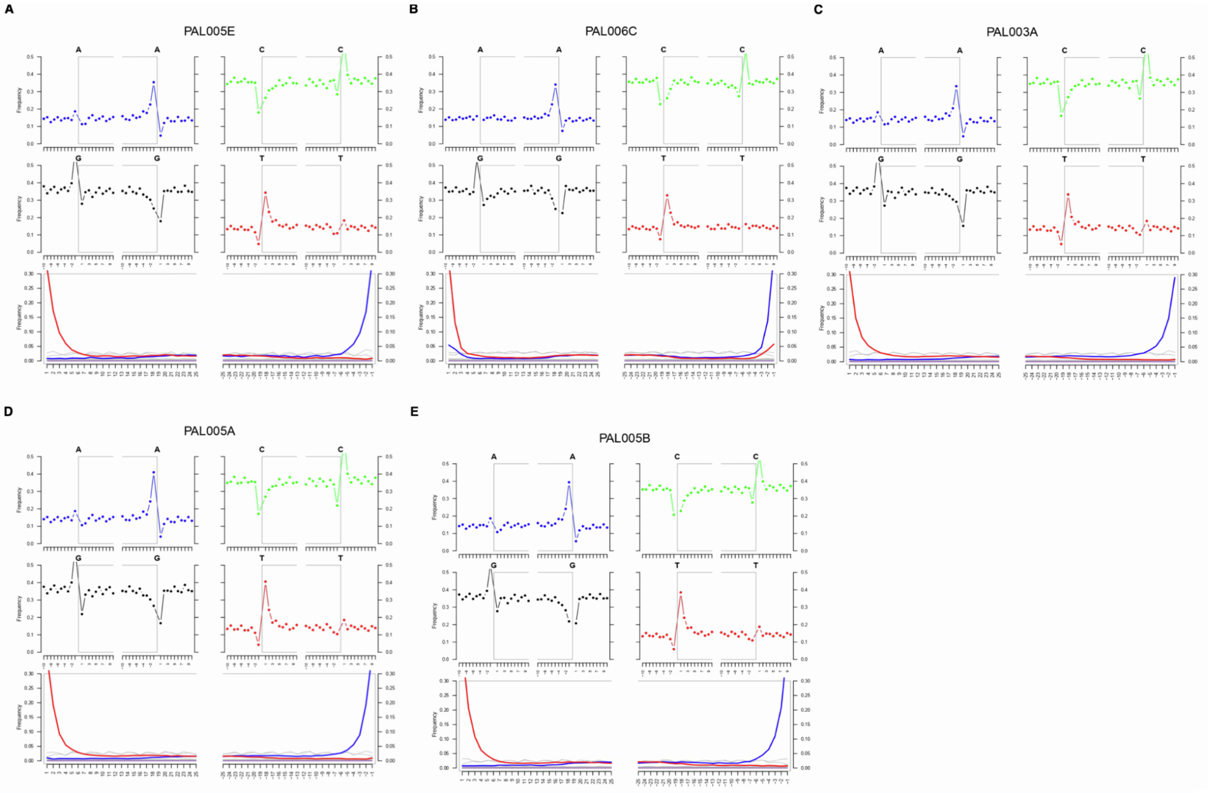

Figure S4. Mis-incorporation plots produced by mapDamage 2.0 after alignment of target samples to Actinomyces sp. oral taxon 414 reference genome. A) PAL005E. B) PAL006C. C) PAL003A. D) PAL005A. E) PAL005B. Related to Figure 4 and STAR Methods. Related to STAR Methods.

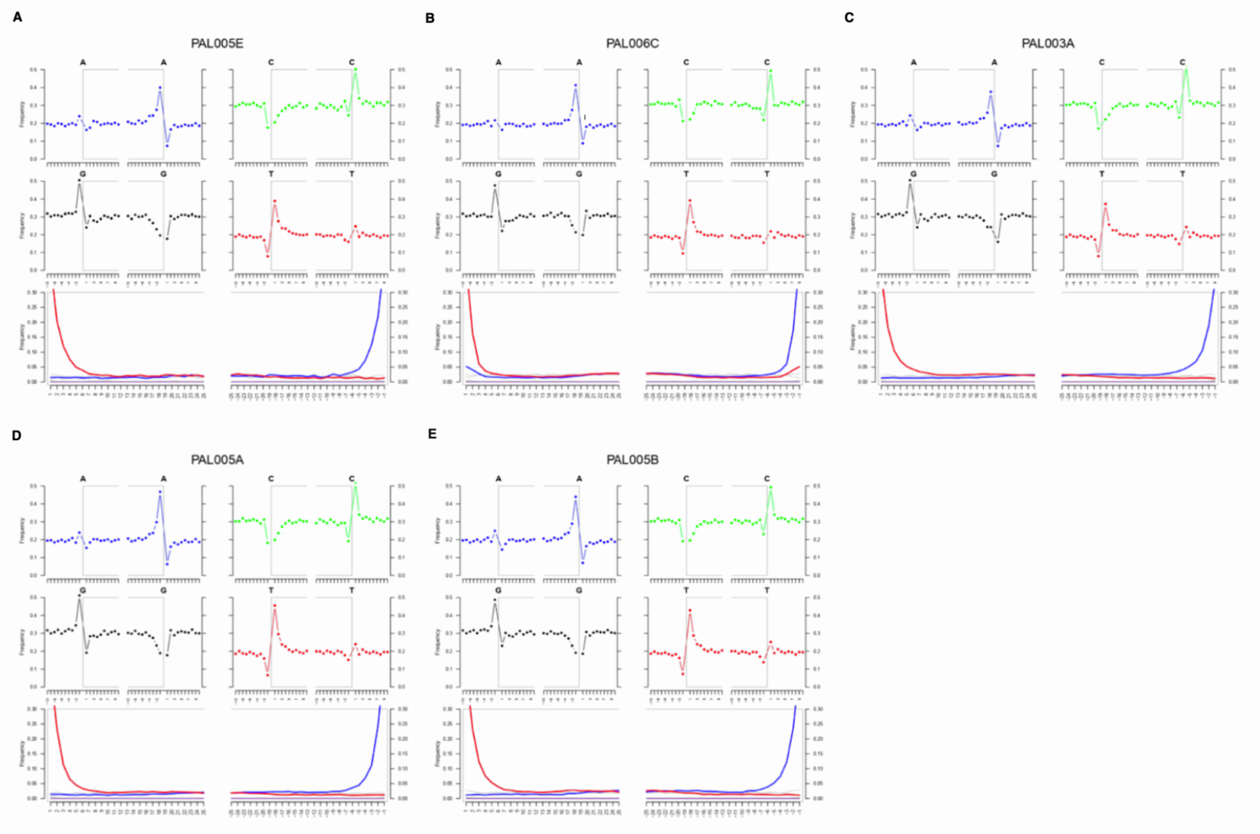

Figure S5. Mis-incorporation plots produced by mapDamage 2.0 after alignment of target samples to Olsenella sp. oral taxon 807 reference genome. A) PAL005E. B) PAL006C. C) PAL003A. D) PAL005A. E) PAL005B. Related to Figure 4 and STAR Methods. Related to STAR Methods.

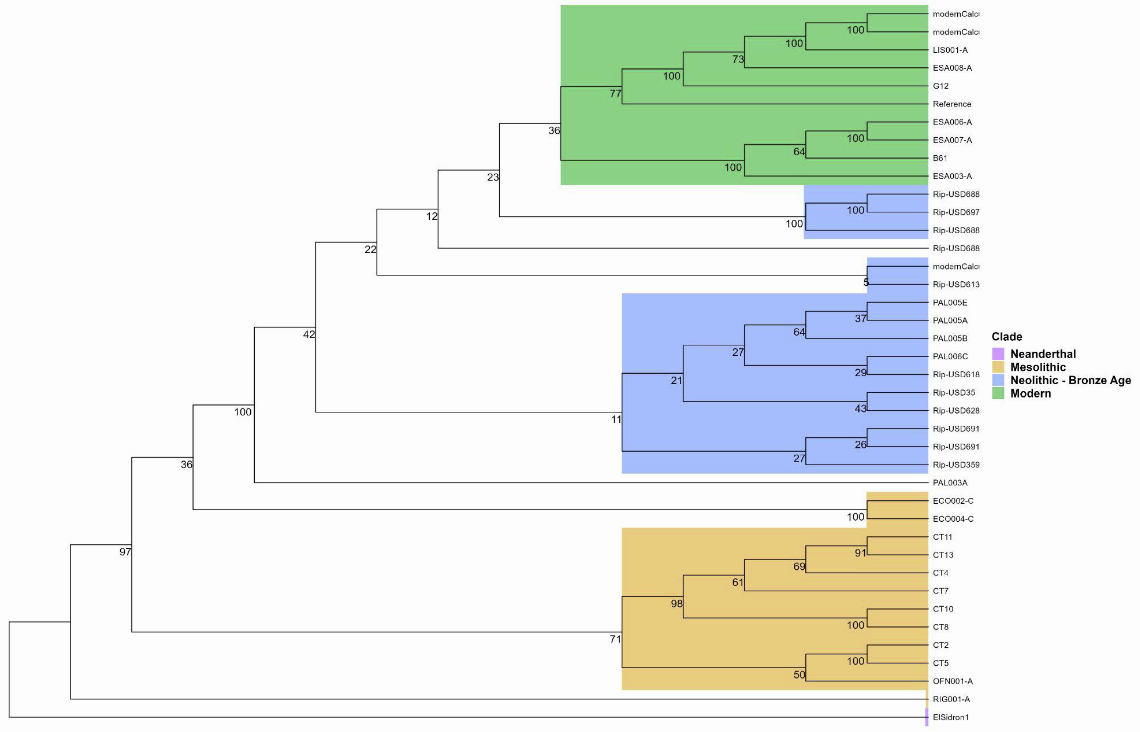

Figure S6. Maximum-likelihood tree created with RAxMl and 1,000 bootstraps. The tree includes 16,469 biallelic positions and 39 modern and ancient Abot 439 strains. Clades are highlighted in colour. Node support is detailed at the base of each node. Branch lengths are not displayed. Related to Figure 4.

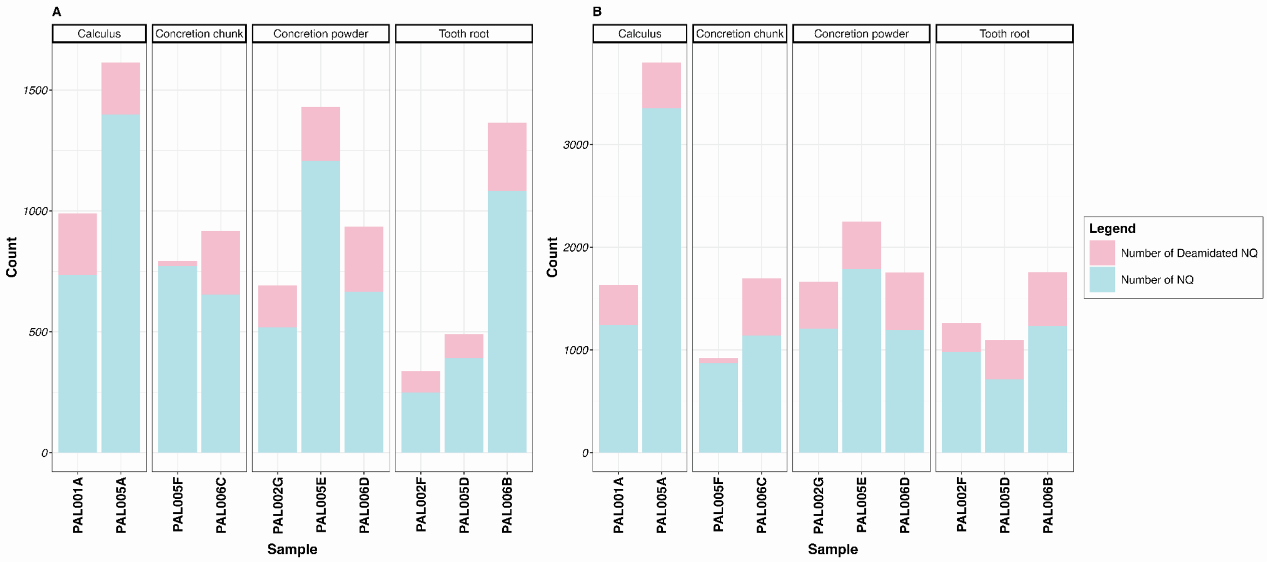

Figure S7. Relative proportions of PTMs per sample type and tool. A) Proportion of NQ deamidated to the number of non-deamidated NQ per sample type with pFind. B) Proportion of NQ deamidated to the number of non-deamidated NQ per sample with novor.cloud. Related to Figure 5.

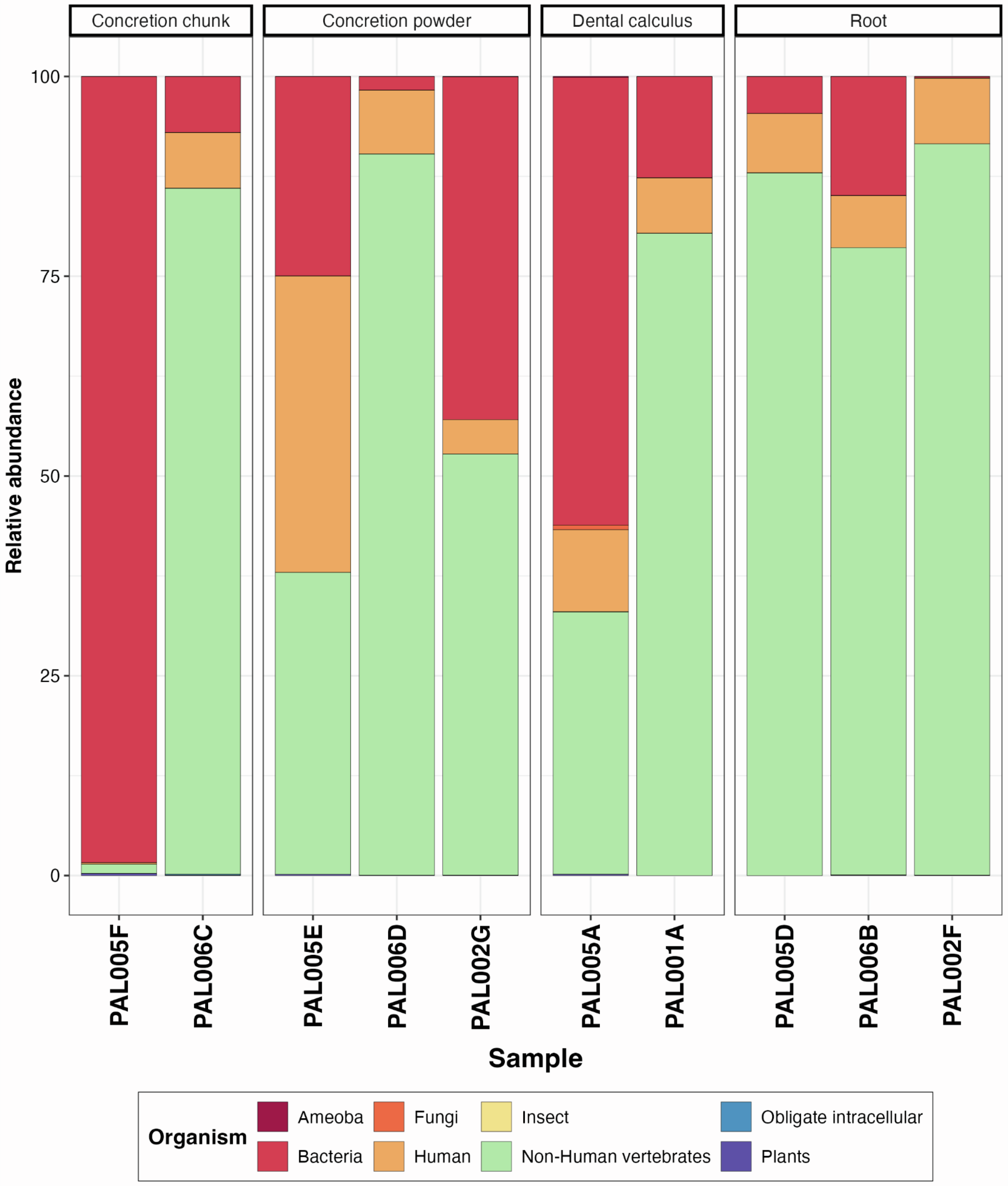

Figure S8. Stacked bar plot showing the relative abundance for peptide origin based on novor.cloud output. Related to STAR Methods.

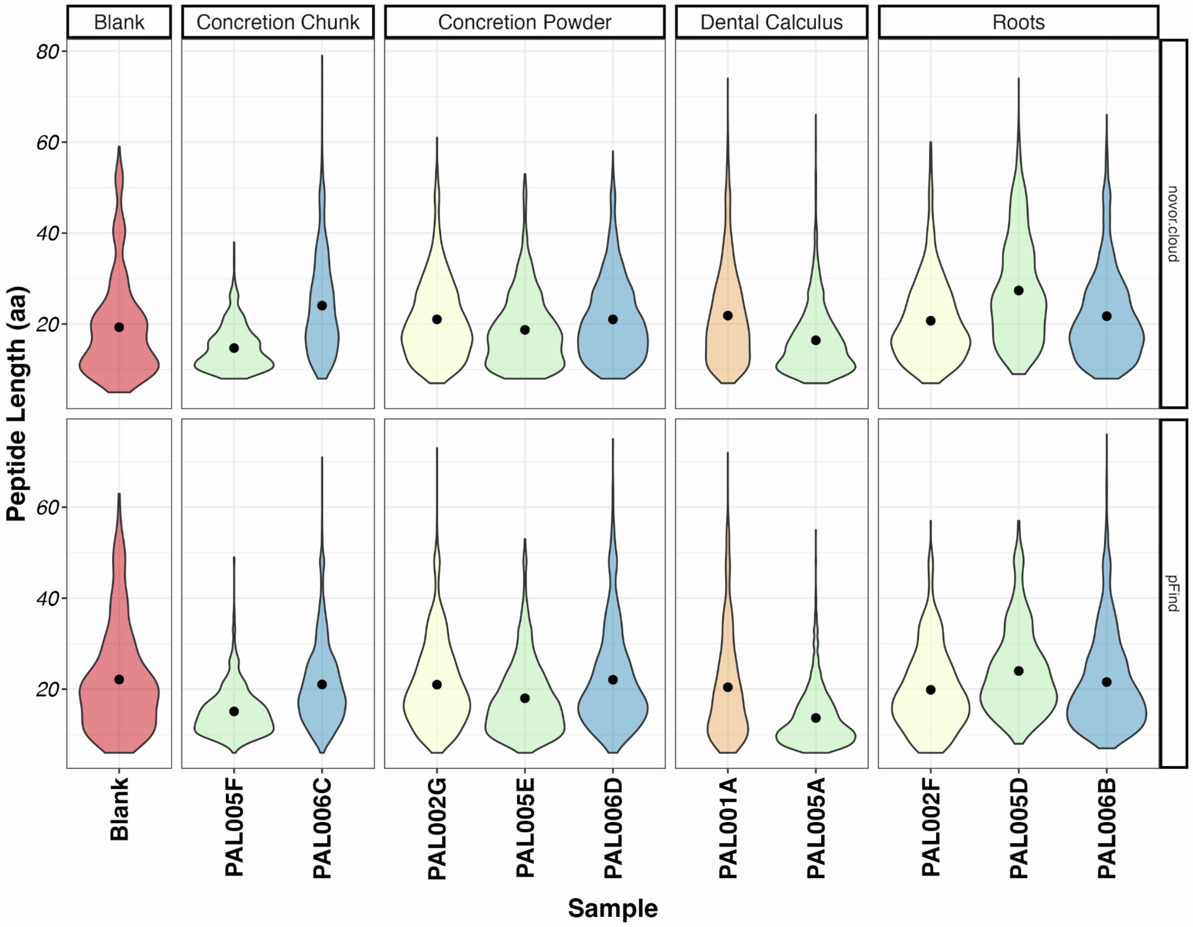

Figure S9. Peptide lengths per sample. Violin plots describing the distribution of peptide lengths (in amino acids), described by novor.cloud and pFind. The colors indicate the different individuals the samples come from, the blank is the extraction blank that was analysed alongside the samples. Related to STAR Methods.

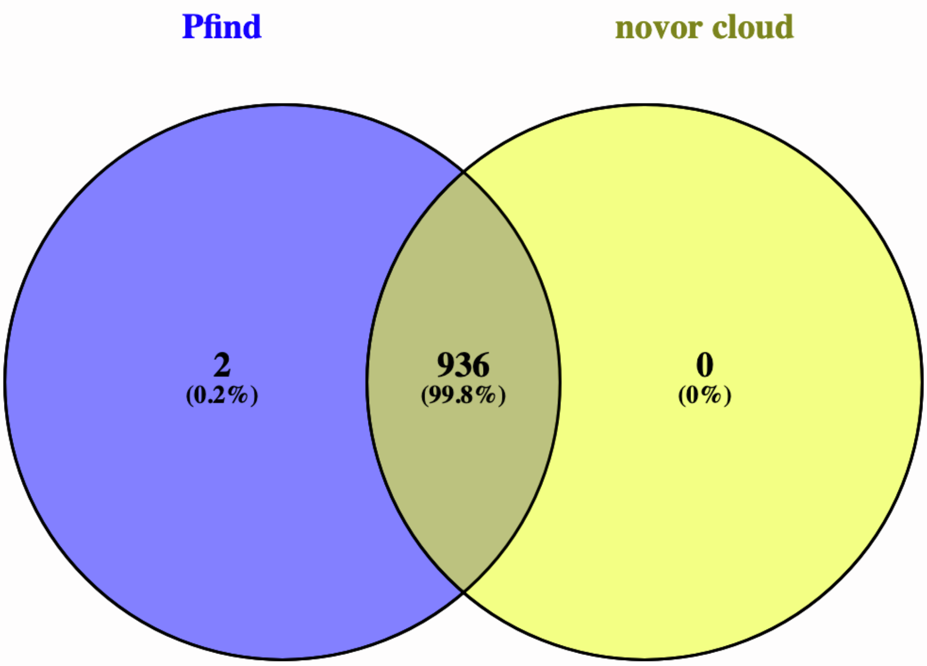

Figure S10. Venn diagram including all unique species identified by novor.cloud and pFind. Related to STAR Methods.

Supplementary Tables

Table S6. Metagenomic sources used for microbial source-tracking, related to Figure 3.

SampleID	Source	Environment	SRA	Publication
modernCalculus-71	Calculus	Oral	ERS3395764	Velsko 2019
modernCalculus-72	Calculus	Oral	ERS3395765	Velsko 2019
modernCalculus-73	Calculus	Oral	ERS3395766	Velsko 2019
modernCalculus-74	Calculus	Oral	ERS3395767	Velsko 2019
modernCalculus-75	Calculus	Oral	ERS3395768	Velsko 2019
modernCalculus-76	Calculus	Oral	ERS3395769	Velsko 2019
modernCalculus-77	Calculus	Oral	ERS3395770	Velsko 2019
modernCalculus-78	Calculus	Oral	ERS3395771	Velsko 2019
modernCalculus-79	Calculus	Oral	ERS3395772	Velsko 2019
modernCalculus-80	Calculus	Oral	ERS3395773	Velsko 2019
ruralGut-10	Human_ruralGut	Human gut	SRR1930145	Rampelli 2015
ruralGut-1	Human_ruralGut	Human gut	SRR1761698	Rampelli 2015
ruralGut-2	Human_ruralGut	Human gut	SRR1761705	Rampelli 2015
ruralGut-3	Human_ruralGut	Human gut	SRR1761710	Rampelli 2015
ruralGut-4	Human_ruralGut	Human gut	SRR1761718	Rampelli 2015
ruralGut-5	Human_ruralGut	Human gut	SRR1761721	Rampelli 2015
ruralGut-6	Human_ruralGut	Human gut	SRR1929408	Rampelli 2015
ruralGut-7	Human_ruralGut	Human gut	SRR1930121	Rampelli 2015
ruralGut-8	Human_ruralGut	Human gut	SRR1930123	Rampelli 2015
ruralGut-9	Human_ruralGut	Human gut	SRR1930141	Rampelli 2015
skin-21	Skin	Skin	SRR1631060	Oh 2016
skin-22	Skin	Skin	SRR1631061	Oh 2016
skin-23	Skin	Skin	SRR1631063	Oh 2016
skin-24	Skin	Skin	SRR1631064	Oh 2016
skin-26	Skin	Skin	SRR3184100	Oh 2016
skin-27	Skin	Skin	SRR3184876	Oh 2016
skin-28	Skin	Skin	SRR3189411	Oh 2016
skin-29	Skin	Skin	SRR3189416	Oh 2016
skin-30	Skin	Skin	SRR3189418	Oh 2016
soil-11	Soil	Soil	ERR671927	Supratim 2023
soil-12	Soil	Soil	ERR671931	Supratim 2023
soil-13	Soil	Soil	ERR671933	Supratim 2023
soil-14	Soil	Soil	ERR671934	Supratim 2023
soil-15	Soil	Soil	ERR671935	Supratim 2023
soil-16	Soil	Soil	ERR671936	Supratim 2023
soil-17	Soil	Soil	ERR671938	Supratim 2023
soil-18	Soil	Soil	ERR687883	Supratim 2023
subPlaque-31	SubPlaque	Oral	SRR061294	HMP 2012

subPlaque-32	SubPlaque	Oral	SRR062298	HMP 2012
subPlaque-33	SubPlaque	Oral	SRR062299	HMP 2012
subPlaque-34	SubPlaque	Oral	SRR513165	HMP 2012
subPlaque-35	SubPlaque	Oral	SRR513768	HMP 2012
subPlaque-36	SubPlaque	Oral	SRR513775	HMP 2012
subPlaque-37	SubPlaque	Oral	SRR514202	HMP 2012
subPlaque-38	SubPlaque	Oral	SRR514239	HMP 2012
subPlaque-39	SubPlaque	Oral	SRR514306	HMP 2012
subPlaque-40	SubPlaque	Oral	SRR514329	HMP 2012
supPlaque-41	SupPlaque	Oral	SRR061192	HMP 2012
supPlaque-42	SupPlaque	Oral	SRR061320	HMP 2012
supPlaque-43	SupPlaque	Oral	SRR061365	HMP 2012
supPlaque-44	SupPlaque	Oral	SRR061562	HMP 2012
supPlaque-45	SupPlaque	Oral	SRR062083	HMP 2012
supPlaque-46	SupPlaque	Oral	SRR063517	HMP 2012
supPlaque-47	SupPlaque	Oral	SRR1804664	HMP 2012
supPlaque-48	SupPlaque	Oral	SRR1804823	HMP 2012
supPlaque-49	SupPlaque	Oral	SRR512767	HMP 2012
supPlaque-50	SupPlaque	Oral	SRR513828	HMP 2012
urbanGut-51	Human_urbanGut	Oral	SRR059389	HMP 2012
urbanGut-52	Human_urbanGut	Oral	SRR059425	HMP 2012
urbanGut-53	Human_urbanGut	Oral	SRR059455	HMP 2012
urbanGut-54	Human_urbanGut	Oral	SRR059917	HMP 2012
urbanGut-55	Human_urbanGut	Oral	SRR060358	HMP 2012
urbanGut-56	Human_urbanGut	Oral	SRR1761677	HMP 2012
urbanGut-57	Human_urbanGut	Oral	SRR1761682	HMP 2012
urbanGut-58	Human_urbanGut	Oral	SRR1761688	HMP 2012
urbanGut-59	Human_urbanGut	Oral	SRR1761692	HMP 2012
urbanGut-60	Human_urbanGut	Oral	SRR1761697	HMP 2012

Supplementary bibliography

1. Quagliati, Q. (1936). La Puglia preistorica (Vecchi & C.).

2. Tinè, S. (1971). La Grotta Scaloria presso Manfredonia. Fasti Archeologici *20*, 175.

3. Gimbutas, M. (1980). Preliminary report on 1979 excavations at Scaloria Cave near Manfredonia, Italy. Dimension sud. Mensile di Politica e Cultura, 12–13.

4. Winn, S.M.M., and Shimabuku, D.M. (1980). The Heritage of Two Subsistence Strategies: Preliminary Report on the Excavations at Grotta Scaloria, Southeastern Italy, 1978 (Department of Anthropology, Saint Mary's University).

5. Robb, J. (2016). Radiocarbon Dating and Absolute Chronology. In The Archaeology of Grotta Scaloria: Ritual in Neolithic Southeast Italy Monumenta Archaeologica., A. Traverso, J. Robb, E. Isetti, and E. S. Elster, eds. (Cotsen Institute of Archaeology Press), pp. 46–56.

6. Robb, J., Elster, E.S., Isetti, E., Knüsel, C.J., Tafuri, M.A., and Traverso, A. (2015). Cleaning the dead: Neolithic ritual processing of human bone at Scaloria Cave, Italy. Antiquity *89*, 39–54.

7. Knüsel, C., Robb, J., and Tafuri, M.A. (2016). The Human Skeletal Remains from Scaloria Cave. In The Archaeology of Grotta Scaloria: Ritual in Neolithic Southeast Italy Monumenta Archaeologica., A. Traverso, J. Robb, E. Isetti, and E. S. Elster, eds. (Cotsen Institute of Archaeology Press), pp. 131–138.

8. Isetti, I., Traverso, A., and Tunzi Sisto, A.M. (2016). Cults and Rites at Scaloria Cave: The Contextual Evidence. In The Archaeology of Grotta Scaloria: Ritual in Neolithic Southeast Italy Monumenta Archaeologica., A. Traverso, J. Robb, E. Isetti, and E. S. Elster, eds. (Cotsen Institute of Archaeology Press), pp. 109–115.
